# Temporal and spatial profiling of *Aedes albopictus* immune responses to chikungunya virus infection

**DOI:** 10.1371/journal.pntd.0013588

**Published:** 2025-10-03

**Authors:** Maria Greta Dipaola, Claudia Fortuna, Francesco Severini, Giulia Bevivino, Marco Di Luca, Tony Nolan, Marco Salvemini, Bruno Arcà, Fabrizio Lombardo

**Affiliations:** 1 Parasitology Unit, Department of Public Health and Infectious Diseases, Sapienza University of Rome, Rome, Italy; 2 Department of Infectious Diseases, National Institute of Health, Rome, Italy; 3 Department of Vector Biology, Liverpool School of Tropical Medicine, Liverpool, United Kingdom; 4 Department of Biology, University of Naples Federico II, Naples, Italy; NIAID: National Institute of Allergy and Infectious Diseases, UNITED STATES OF AMERICA

## Abstract

The global expansion of *Aedes albopictus* from Southeast Asia to various regions worldwide poses a significant public health concern due to its role as a vector for several pathogens, including chikungunya virus (CHIKV), which infects over one million people annually. In this study, aimed at understanding the molecular interactions between *Ae. albopictus* and CHIKV, we analyzed by RNA sequencing CHIKV-infected and uninfected control mosquitoes. We focused our attention on key mosquito organs at one- and five-days post-blood meal ingestion, which correspond to viral entry and dissemination, and found specific transcriptional changes involving various pathways during the CHIKV lifecycle. The mosquito midgut plays a crucial role in the early stages, when the virus enters along with human blood components, encounters the resident microbiota, interacts with the developing peritrophic matrix, and counteracts the mosquito’s digestive enzymes. We found that RNA interference (RNAi) was predominantly activated in the midgut during the initial virus invasion. Additionally, several key enzymes involved in autophagy and ubiquitination were also more abundant in infected midguts compared to controls. At later time points, after viral dissemination into the hemocoel, key immune responses are triggered in the hemolymph and, accordingly, immune mechanisms such as the activation of leucine-rich repeats (LRRs) proteins, secretion of antimicrobial peptides (e.g., holotricin), and melanization (mediated by phenoloxidase, PO) were the most prominent. RNA-seq results were validated by RT-qPCR on selected candidates in different tissues and a catalogue of *Ae. albopictus* immune genes (891 contigs) grouped into 24 different immune and immune-related families was compiled. This study explores the molecular interactions between *Ae. albopictus* and CHIKV across developmental stages, providing key insights into arbovirus transmission dynamics and mosquito vector competence.

## Author summary

The tiger mosquito, *Aedes albopictus*, has emerged as a significant public health threat due to its role in transmitting several arboviruses, such as the chikungunya virus (CHIKV), which affects millions of people worldwide. In this study, we investigated how mosquito gene expression is modulated during key stages of the CHIKV life cycle using RNA sequencing. Our analysis targeted critical mosquito tissues and organs at one- and five-days post-blood meal ingestion, corresponding to the viral phases of entry in the midgut and of systemic spread in the hemolymph, respectively. We identified distinct transcriptional patterns associated to the different stages of CHIKV infection, depicting dynamic host-pathogen interactions that are critical in shaping vector competence—the ability of a mosquito species to acquire, maintain, and transmit a specific pathogen. Understanding these molecular mechanisms may advance our knowledge of mosquito antiviral defenses and support the development of more targeted and effective vector control strategies.

## Introduction

The global spread of *Aedes albopictus*, a highly aggressive, daytime-biting mosquito known for transmitting several human-pathogenic arboviruses, represents a significant public health challenge. Commonly referred to as the Asian Tiger mosquito, *Ae. albopictus* is recognized as one of the 100 worst invasive species worldwide (Invasive Species Compendium, 2001). It has rapidly expanded beyond its native Southeast Asia range to other countries, demonstrating remarkable ecological adaptability in traits such as feeding behavior, diapause, and vector competence [1–5]. *Aedes albopictus* can transmit a variety of arboviruses, including *Flaviviridae* (*Orthoflavivirus*) as dengue virus (DENV), West Nile virus (WNV), Zika virus (ZIKV), and *Togaviridae* (*Alphavirus*) as Sindbis virus (SINV), and chikungunya virus (CHIKV). While CHIKV is primarily spread by *Aedes aegypti*, a viral RNA mutation in 2006 enabled *Ae. albopictus* to also become a highly competent vector [6]. CHIKV has spread extensively, infecting over a million people annually and causing significant health problems globally [7–9]. Over the past 15 years, following sporadic outbreaks, the virus has caused significant epidemics in areas including Africa, Asia, the Indian Ocean, Europe (notably with two outbreaks in Italy in 2007 and 2017), and more recently, the Caribbean and the Americas [10,11].

Molecular interactions between mosquitoes and arboviruses begin once the mosquito acquires an infected blood meal. After entering the mosquito body, the virus follows a mandatory pathway, which can span from a few days to several weeks and requires the overcoming of various anatomical and immune barriers for the successful transmission to a new host [12]. Although mosquitoes lack adaptive immunity, their defense mechanisms against pathogens are highly organized and occur at multiple levels. The first line of defense consists of several physical barriers that prevent pathogen invasion, including the cuticle, midgut, hemocoel, and salivary glands [13,14]. Beyond these tissue barriers, mosquito also mount immune responses that involve both humoral and cellular components and are mediated by mosquito hemocytes and fat body cells [15]. After the ingestion of an infected blood meal, the virus reaches the mosquito midgut, where it encounters the peritrophic matrix, composed by proteolytic enzymes that digest the blood meal, and it faces an early immune response (around one day post-infection, dpi). Following a significant reduction in viral particles, the virus invades the epithelial cells, where it replicates and begins its escape from the midgut compartment to spread in the hemocoel. There, a second later immune response is triggered, involving mosquito immune cells such as hemocytes and fat body cells. Finally, the virus eventually invades the salivary glands (between 5 and 10 dpi), where it replicates again before being transmitted to a new host [12].

The first step in the mosquito immune response is pathogen recognition, which occurs through interactions between pattern recognition receptors (PRRs) and pathogen-associated molecular patterns (PAMPs). PRRs are host-secreted molecules located in various compartments of the mosquito body, such as the midgut lumen and hemocoel. These receptors have adhesive domains that can detect and bind to PAMPs, which are structural or surface components of pathogens [16]. Key PRRs include: fibrinogen-related proteins (FREPs), which are primarily involved in immune responses against bacteria [17]; Toll-like receptors [18] and thioester-containing proteins (TEPs), which play a role in pathogen neutralization [19]; leucine-rich repeat (LRR) proteins, which also participate in ligand-receptor interactions and immune defenses [20]. Once the pathogen is recognized, the immune system activates various cellular and humoral responses through signaling pathways such as the Imd, Toll, JAK-STAT and RNAi pathways. Activation of humoral immune pathways stimulates the production and secretion of antimicrobial peptides (AMPs), which are produced by midgut epithelial cells and immune cells into the hemolymph [21,22] in response to bacterial and viral infections [14,23,24]. The involvement of various immune pathways in combating arboviral infections has been extensively documented. For instance, studies on *Ae. aegypti* mosquitoes infected with the DENV revealed that activation of the Jak-STAT pathway has a relevant role in controlling DENV replication [24]. Similarly, the Toll-like pathway has been implicated in the defense against both DENV and ZIKV in *Ae. aegypti*, although it showed minimal impact on CHIKV infection [14]. Also, the Imd pathway, which was initially thought to play a minor role in antiviral defense against DENV, CHIKV, and ZIKV [25–27], seems to play a role in limiting viral replication, as suggested by studies in *Ae. aegypti* [15,28]. Moreover, a cecropin-like antibacterial gene was found upregulated in the salivary glands of *Aedes* mosquitoes following infection with DENV and CHIKV, likely through the activation of the Imd pathway [29,30]. However, despite the importance of these pathways, the RNA interference (RNAi) mechanism, which detects virus-derived double-stranded RNA (dsRNA) and triggers the production of small interfering RNAs (siRNAs), is recognized as the primary mosquito defense strategy against various viruses, including CHIKV and ZIKV [25,31,32]. Additionally, also the classical microRNA (miRNA) pathway seems to play a role in the regulation of mosquito immune responses and in antiviral defense [33–36] as shown in *Ae. aegypti* mosquitoes infected with WNV, DENV, and CHIKV [37–39].

Cellular responses to viral infection in mosquitoes involve several processes primarily mediated by hemocytes and fat body cells and include apoptosis, autophagy, phagocytosis and melanization. Studies on *Ae. aegypti* mosquitoes infected with SINV demonstrated that apoptosis plays a complex role in viral dynamics, suggesting that apoptosis may function as a mechanism that supports, rather than suppresses, viral infection in such context [40]. Autophagy, on the other hand, can be triggered by various stimuli, including innate immune signals and cellular stress. Viral infections frequently induce autophagic responses, which can serve as a defense mechanism against viral replication [41]. This antiviral process is facilitated by the autophagy cargo receptor p62, which identifies viral capsid proteins tagged with polyubiquitin chains. Then, p62 transports these proteins to autophagosomes for degradation [42]. Consequently, the ubiquitination of viral proteins plays a critical role in viral control and may be an essential component of the mosquito’s immune response to invading pathogens. Other cellular responses contribute to the mosquito defence against viruses, though to a lesser extent, as indicated by various studies. Hemocyte-mediated melanization via the prophenoloxidase (PPO) cascade and phagocytosis may take place in the mosquito hemocoel in response to viral diffusion. These processes not only reduce arboviral replication and systemic dissemination but also restrict the mosquito capacity to transmit arboviruses [43–45].

Most studies on mosquito antiviral immunity have concentrated on *Ae. aegypti*, leaving a notable gap in our understanding of immune responses in other *Aedes* species. Few transcriptomic studies have been conducted on *Ae. albopictus*, and only one on *Aedes malayensis*. Early transcriptomic reports investigating the molecular interactions occurring between *Ae. albopictus* and CHIKV focused on infected midguts at 2 dpi and thoraxes at 8 dpi. In the midgut at 2 dpi, only a small number of differentially expressed genes (DEGs) were identified, mainly related to metabolism, with no genes linked to immunity [46]. In contrast, significant transcriptomic changes were observed in *Ae. albopictus* mosquitoes following CHIKV dissemination in the head and thorax, a region containing the salivary glands [47]. Another study involved a high-throughput transcriptomic analysis of *Ae. albopictus* and *Ae. malayensis* infected with DENV and CHIKV. This study analyzed dissected midguts at two early time points (1 and 4 dpi), uncovering a complex pattern of transcriptomic changes, particularly immune regulations, with different immune pathways activated by arboviral infection [48].

The primary objective of the present study was to investigate temporal and spatial dynamics of the molecular interactions that occur during the CHIKV lifecycle within the Asian tiger mosquito, *Ae. albopictus*. Specifically, we investigated how immune and defence pathways are regulated at two key stages of infection and in different mosquito tissues: (i) one day post-infection (dpi), corresponding to midgut invasion, and (ii) five dpi, when the virus disseminates into the hemocoel. To this end, we employed an RNA-seq approach to compare CHIKV-infected and uninfected *Ae. albopictus* mosquitoes by analysing midguts at both 1 and 5 dpi, and carcasses (the remainder of the body excluding the midgut) at 5 dpi. Additionally, whole uninfected and infected females were analyzed at both 1 and 5 dpi. To validate the expression patterns of selected candidate genes, we performed RT-qPCR on dissected midguts and isolated circulating hemocytes to assess tissue-specific gene expression. Lastly, we updated and expanded the existing *Ae. albopictus* immune gene catalogue [48], leveraging the recently released AalbF5 genome assembly. By examining the local and systemic molecular interactions between *Ae. albopictus* and CHIKV at various developmental stages, this study offers valuable insights in the understanding of virus transmission dynamics and provides essential knowledge for developing new strategies to reduce mosquito vector competence.

## Materials and methods

### Mosquitoes, infections, and tissue dissections

Mosquitoes (*Ae. albopictus* Roma-RM strain mosquitoes, collected and reared in Rome, Italy) were reared under standard laboratory conditions (25 ± 1 °C, relative humidity 60 ± 10%, light: dark photoperiod 14:10 h) in the insectary of the Department of Infectious Diseases at the National Institute of Health, Rome (Istituto Superiore di Sanità, ISS). In vivo experimental infections of *Ae. albopictus* mosquitoes with CHIKV were performed in a biosafety level 3 laboratory (BSL3) at the ISS, obtaining three independent biological replicates. In each experiment, around 120–150 females 5–7 dpe (days post emergence) were collected to allow infected blood feeding (rabbit blood with viral particles of the CHIKV strain isolated from a human biological sample collected during the Italian 2007 CHIKV outbreak [49]; titer: 2.98x10^7^/ml) and control blood feeding (rabbit blood with MEM containing inactivated fetal serum, amino acids, penicillin, streptomycin). After blood feeding (1 hour lasting), mosquitoes were chilled on ice and engorged females were isolated. Mosquitoes were then dissected at two different timepoints (T), 1 day (T1) and 5 days (T5) post infected blood meal (dpi): for each replicate, around 20 midguts and 6–8 carcasses (whole body without the midgut) were collected for each T as well as 6–8 whole females (details are reported in the S1 Text and Table A in S1 Text). The samples were next collected in RNA later and then stored at -80°C until further use.

### RNA extraction, library preparation and sequencing

Total RNA was extracted from midguts, carcasses, and whole bodies of infected and not-infected female mosquitoes at different T (T1 and T5), quantified by spectrophotometric reading (Take3 module of the Microplate Reader BioTek SynergyHT) and evaluated by agarose gel electrophoresis. Only samples showing appropriate quality and quantity parameters were selected for further treatments, i.e., DNase I treatment, library preparation and RNA-seq. Before proceeding to NGS (Next Generation Sequencing), CHIKV titer was also determined in representative samples by Real Time quantitative PCR (RT-qPCR). To evaluate CHIKV titer, 3–5 entire females and 8–10 carcasses (females after midguts dissections) were collected at day 5 and analysed by RT-qPCR (see below for technical details and Supplementary S1 Text): all the samples analysed confirmed the occurrence of viral infection in the mosquitoes. RNA samples were initially quantified using the Qubit 3.0 Fluorometer to ensure that the quantity of material submitted was adequate. Total RNA integrity was checked using the Fragment Analyzer (Bioanalyser) to measure the samples quality as RNA Quality Number (RQN). RNA-seq was performed at Polo d’Innovazione di Genomica Genetica e Biologia SCaRL, Siena – Italy, thanks to a grant awarded by the European Project Infravec2 (http://infravec.mdmdemo.ch). The libraries were prepared in accordance with the Illumina TruSeq Stranded mRNA Sample Preparation Guide for Illumina Paired-End Indexed Sequencing and then validated using the Fragment Analyzer to check the distribution. Finally, concentration of library samples was defined based on the Qubit 3.0 Fluorometer quantification. Indexed DNA libraries were normalized to 2 nM and then pooled in equal volumes. The pool was loaded at a concentration of 1.2 pM onto an Illumina NextSeq 550 Flowcell High Output, with 1% of Phix control. The samples were then sequenced using the Illumina chemistry V2, 2x75 bp paired end run.

### Expression profiling and identification of differentially expressed transcripts

Raw sequencing reads were subjected to quality filtering using Trimmomatic-0.32 to eliminate low-quality sequences and adaptor contaminants [50]. The resulting high-quality read pairs were aligned to the *Ae. albopictus* reference transcriptome (Genome version: AalbF5, assembly: GCF_035046485.1, NCBI) using Bowtie [51]. Expression levels were quantified as Fragments Per Kilobase of transcript per Million fragments mapped (FPKM) using RSEM [52] (Supplementary S1 Data). Differential expression analysis was performed using edgeR and pairwise comparisons between the conditions [53]. Transcripts with a False Discovery Rate (FDR) less than 0.05 and a Fold Change (FC) greater than 2 were considered statistically significant and selected for subsequent analyses. DE contigs were identified for the following comparisons (Supplementary S2 Data): infected midguts (Mi-V) versus control midguts (Mi-C) at T1; both infected midguts (Mi-V) versus control midguts (Mi-C), and infected carcasses (Ca-V) versus control carcasses (Ca-C) at T5.

### Pfam and GO enrichment

To identify enriched Gene Ontology (GO) and Protein Families (Pfam) terms, enrichment analyses were conducted for the following pairwise comparisons: at T1, infected midguts (Mi-V) versus control midguts (Mi-C); and at T5, both infected midguts (Mi-V) versus control midguts (Mi-C), and infected carcasses (Ca-V) versus control carcasses (Ca-C) (Supplementary S3 Data and S4 Data). The sma3s and pfamscan software [54–56] were utilized to assign, respectively, GO terms and Pfam term to transcripts of the AalbF5 reference transcriptome and to transcripts identified as differentially expressed by edgeR analysis in pairwise T1 and T5 comparisons. Subsequently, Fisher’s Exact Test was applied to assess the overrepresentation of specific GO terms and Pfam domains among the differentially expressed transcripts, with p-values adjusted via the Benjamini-Hochberg procedure to control for multiple testing (significance threshold: adjusted p-value < 0.05) [57]. All statistical analyses were conducted within the R programming environment [58].

### Validation of candidate genes expression by quantitative real-time PCR

To validate the transcript abundance patterns identified through RNA-seq analysis, RT-qPCR was conducted on five selected candidates. Tissues and organs were dissected from uninfected adult females (4–7 days post-emergence, dpe), which were reared under standard laboratory conditions in the Insectary of the Department of Public Health and Infectious Diseases at Sapienza University and maintained on a 10% sucrose diet. Mosquitoes were initially anesthetized on ice for 2–3 minutes, and dissections were carried out to obtain three independent biological replicates for each of the following tissues/organs: hemolymph (collected using the proboscis clipping technique and containing only circulating hemocytes), ovaries, midguts, carcasses without hemolymph, carcasses without the midgut, carcasses without the ovaries and head, and whole females. The samples were then preserved in PBS solution at -80°C until RNA extraction.

Total RNA was extracted from the collected samples using Trizol reagent (Invitrogen), following the manufacturer’s instructions. The RNA quantity and quality were assessed through spectrophotometric measurements (using the Take3 module of the BioTek SynergyHT plate reader with GEN5 software) and 1% agarose gel electrophoresis. To eliminate genomic DNA contamination, DNase I treatment was performed using the Ambion DNA-free kit, following the manufacturer’s protocol. The efficiency of DNase I treatment was verified by endpoint PCR, comparing untreated and treated RNA samples using the ribosomal S5 (rpS5) gene as a target. Different amounts of DNase I-treated RNA (ranging from 200 ng to 1 µg) were used as templates to synthesize First-Strand cDNA with Superscript II RT (Invitrogen) and Oligo dT (Invitrogen), following the manufacturer’s guidelines. The resulting cDNA samples were diluted to a concentration of 10 ng/µL and used as templates for quantitative real-time PCR. Standard curves were generated for each target gene as well as for the endogenous reference gene (rpS5). Specifically, the cDNA samples used for standard curve preparation were derived from a pool of four whole female mosquitoes, following the previously described procedures. Serial dilutions of cDNA were prepared to establish five points for each standard curve (dilution factor: 1:5) at the following concentrations: [100 ng/µL], [20 ng/µL], [4 ng/µL], [0.8 ng/µL], and [0.16 ng/µL]. Each PCR reaction began with an initial holding stage of 2 minutes at 50 °C, followed by 2 minutes at 95 °C. This was followed by 40 cycles of amplification (95 °C for 15 seconds; 60 °C for 1 minute). A melt curve analysis was included to evaluate primer efficiency and confirm the specificity of the amplicon for each target gene. Relative quantification analysis was performed using both the standard curve method and the ΔΔCt method, where applicable. In the standard curve approach, the relative expression levels of selected genes across different tissues were determined by calculating the ratio of the target gene to the endogenous reference gene (rpS5), based on their Ct values interpolated from the standard curves. Statistical analysis was conducted using one-way ANOVA, followed by Tukey’s multiple comparisons test, applied to log10-transformed values from the three biological replicates. Statistical significance was denoted as follows: *p < 0.05; **p < 0.01; ***p < 0.001. Primers’ sequences are reported in Supplementary S1 Text (Table D in S1 Text).

## Results

### Experimental outline

**Fig 1:**
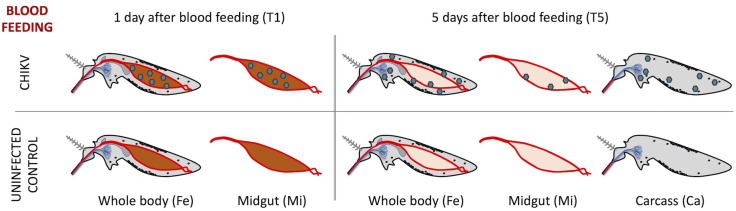
Schematic representation of the experimental design and biological samples used for RNA-seq. The diagram illustrates key mosquito tissues sampled for RNA sequencing. Depicted within the mosquito body are: the gut (midgut shown in dark red at T1 and light red at T5), the crop (dark grey diverticulum connected to the foregut), the salivary glands (light blue structures in the thorax), hemocytes and fat body cells (represented by black dots in the grey hemolymph), and virions (blue hexagons). The term carcass refers to the entire body after the midgut has been removed.

The main aim of this study was to better understand the *Ae. albopictus* responses to CHIKV infection, focusing specifically on two key stages of the viral lifecycle within the mosquito: the early phase, when the virus enters the mosquito midgut after an infectious blood meal, and the later stage, when the viral particles cross the midgut and spread into the mosquito haemolymph. In fact, shortly after ingestion with the blood, at 1 day post-infection (dpi), the virus is initially targeted by the “midgut infection barrier,” an immune response primarily driven by midgut epithelial cells and/or midgut-associated hemolymph cells [13,59]. During this early phase, several additional factors may influence the outcome of the infection, including abundance and composition of the mosquito microbiota, production of the peritrophic matrix and effectiveness of blood digestion. Viral particles eluding this first line of defense may replicate within midgut cells, cross the “midgut escape barrier,” and disseminate into the mosquito hemocoel [60]. This triggers a second wave of immune responses, which take place in the few days following midgut escape and primarily involves cellular components of the hemolymph (such as hemocytes, fat body cells, and other immune cells) working together to counteract viral replication, diffusion and invasion of salivary glands [14]. The balance between the mosquito immune responses and viral replication and dissemination is central in determining the mosquito competence for transmitting the virus to the next host. Consequently, our experimental design employed an RNA-seq approach to analyze the transcriptional responses of infected and uninfected mosquitoes at 1 and 5 dpi, with a special focus on midgut, as representative of the early responses to infection and on carcasses (whole body without midguts, i.e., containing hemolymph) as representative of later responses following viral dissemination (Fig 1).

### Sequencing, transcriptome assembly and annotation, and differential expression (DE) analysis

Total RNA was extracted from the samples summarized in Fig 1 and used for library preparation and sequencing. A total of 1,170,016,748 Illumina raw reads was generated (Table 1) across 24 libraries (with two or three biological replicates for each time point). Raw RNA-seq sequences were deposited in the NCBI Sequence Read Archive (SRA) database with accession number PRJNA1260742. After trimming adapters and filtering out low-quality reads, cleaned reads were mapped against the reference AalbF5 transcriptome to calculate row counts and expression values for each of the 38,020 transcripts, using the FPKM metrics (Supplementary S1 Data).

**Table 1:** Summary of samples and RNA-Seq Data.

Sample	Tissue	Blood feeding	Replicate	PE reads number
T1 1 day after blood feeding	Fe	C	T1C_F1	40732234
			T1C_F3	39964324
	Mi	C	T1C_M1	45403250
			T1C_M2	40663238
	Fe	V	T1V_F1	40969340
			T1V_F2	38590204
	Mi	V	T1V_M1	44240456
			T1V_M2	41195324
T5 5 days after blood feeding	Fe	C	T5C_F1	46571134
			T5C_F2	50713868
			T5C_F3	60399568
	Mi	C	T5C_M1	48152700
			T5C_M3	62415816
			T5C_M4	43592708
	Ca	C	T5C_C1	53019206
			T5C_C2	58974976
			T5C_C3	67598474
	Fe	V	T5V_F2	78021952
			T5V_F3	37850892
	Mi	V	T5V_M1	42292572
			T5V_M2	52737534
			T5V_M4	44390582
	Ca	V	T5V_C2	56320238
			T5V_C3	35206158

Time points following either infective (V) or non-infective (C) blood meals are indicated, along with the tissues used for RNA-seq analysis (including replicates). Fe refers to the whole female body; Mi to the midgut; and Ca to the carcass (whole body excluding the midgut). C denotes mosquitoes fed on an uninfected blood meal (control), while V indicates mosquitoes fed on a CHIKV-infected blood meal. PE, Paired-End Read Counts.

To identify differentially expressed (DE) contigs, datasets from infected and uninfected control samples—specifically, midguts at T1 and carcasses at T5—were compared using edgeR tool (Table 2).

**Table 2:** Summary of Differential Expression (DE) Analysis. The table reports the number of upregulated (UP) and downregulated (DOWN) genes at three significance thresholds (FDR < 0.05, FDR < 0.01, FDR < 0.001). Comparisons shown are: Mi T1 V vs C (midguts at 1 dpi, CHIKV-infected vs control) and Ca T5 V vs C (carcasses at 5 dpi, CHIKV-infected vs control).

	FDR < 0.05		FDR < 0.01		FDR < 0.001	
	UP	DOWN	UP	DOWN	UP	DOWN
Mi T1 V vs C	73	72	39	44	22	17
Ca T5 V vs C	41	85	24	51	9	22

**Fig 2:**
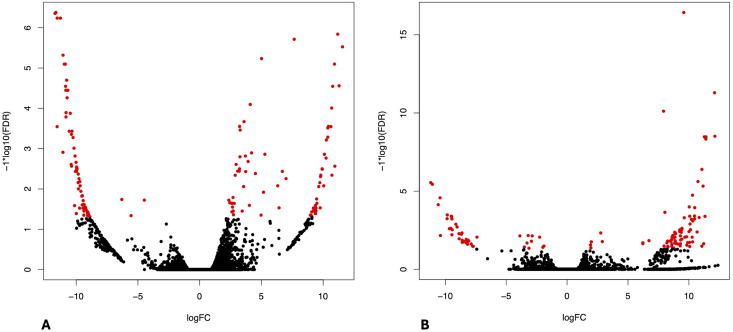
Volcano plots displaying the results of the differential expression (DE) analysis. **(A)** DE contigs in midguts at T1. **(B)** DE contigs in carcasses at T5. The x-axis represents Log10 fold change (FC), while the y-axis represents -Log10 false discovery rate (FDR). DE genes are highlighted in red. Transcripts differentially upregulated in the infected groups compared to control groups are shown on the left side of each graph, whereas those downregulated in the infected groups compared to control groups are displayed on the right side.

Differential expression analysis revealed that at day 1 post-infection (T1) 73 contigs were upregulated in CHIKV-infected midguts, while 72 were downregulated in CHIKV-infected midguts compared to the control group with FDR ≤ 0.05 (Fig 2A). Similarly, at day 5 post-infection (T5), 41 contigs were more abundant in CHIKV-infected carcasses and 85 were downregulated in CHIKV-infected carcasses compared to the control group (Fig 2B). The whole edgeR outputs, including the comparison between infected and control midgut at 5 dpi, are reported in Supplementary S2 Data.

A minimal overlap was observed when comparing the different DE groups summarized in Table 2 (see Fig B and Table C in S1 Text). This low degree of overlap suggests that distinct, organ-specific responses are activated within the mosquito compartments (midguts and carcasses) during the different stages of the virus lifecycle: at 1 day post-infection, when the virus enters the enterocytes, and at 5 days post-infection, when the virus disseminates into the mosquito hemocoel. Such limited overlap in transcriptionally modulated *Ae. albopictus* genes upon CHIKV infection across different organs (midguts vs. carcasses) and time points (1 and 5 days post-infection) was also observed in previous studies by Vedururu and colleagues [46,47], and by Modahl and colleagues [48].

We also performed GO and Pfam enrichment analyses by comparing GO terms (encompassing the three main categories: biological process, BP; cellular component, CC; molecular function, MF) and Pfam terms for each sample against the general transcriptome. Differentially enriched GO and Pfam terms in infected and control midguts at day 1 (T1_Mi_C-UP and T1_Mi_V-UP), and infected and control carcasses at day 5 (T5_Ca_C-UP and T5_Ca_V-UP) are shown in the following figures and described in more detail below for each timepoint. Analysis of infected and control midguts at day 5 post-infection (T5_Mi_C-UP and T5_Mi_V-UP) are reported in Supplementary S1 Text (Table B and Fig A in S1 Text). To complement the information provided by GO and Pfam enrichment analyses, selected DE contigs for each group have also been included in the figures (for complete DE, GO and Pfam lists, refer to the Supplementary S2 Data, S3 Data and S4 Data and Supplementary S1 Text).

### Gene expression profiles in midguts at day 1 post infection

As already mentioned, the midgut epithelium is the first physical barrier the virus encounters during its lifecycle within the mosquito. Various factors contribute to the complex interactions between the virus and the midgut lumen environment, including the microbiota, the formation of the peritrophic matrix, and the secretion of proteolytic enzymes responsible for blood meal digestion. Moreover, virus invasion and replication within midgut cells evokes specific defense responses. Among these responses, the RNAi pathway is known to be activated by CHIKV during the early stages of its lifecycle in the mosquito midgut.

**Fig 3:**
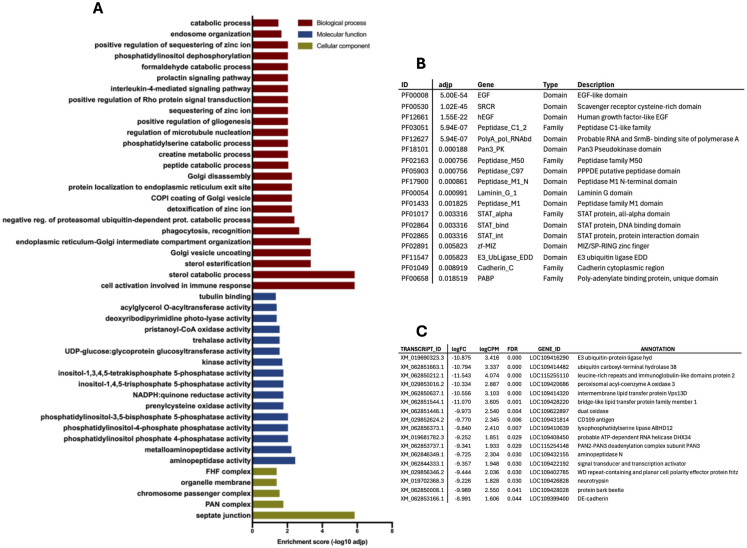
Selection of enriched terms in infected midguts at 1 dpi, i.e., upregulated in response to CHIKV infection. **(A)** List of enriched Gene Ontology (GO) terms according to the three categories, Biological process (BP, red bars), Molecular Function (MF, blue bars) and Cellular Component (CC, green bars); **(B)** list of enriched Pfam domains, families and repeats: Pfam IDs and descriptions are reported as the adjusted p value (adjp); **(C)** list of differentially expressed (DE) contigs: transcript IDs, according to AalbF5 genome assembly, are reported with relative annotation; enrichment parameters are logFC (Fold Change) logCPM (Counts Per Million) and FDR, False Discovery Rate.

Accordingly, several differentially expressed (DE) transcripts and Pfam domains linked to RNA interference (RNAi)-mediated defense mechanisms were significantly more abundant in infected midguts. These included RNA helicases and subunits of the PAN2-PAN3 deadenylase complexes [61–63]. Additionally, other upregulated GO terms, Pfam categories, and DE contigs in virus-infected midguts (Fig 3) were related to ubiquitination processes, such as E3 ubiquitin ligases and ubiquitin hydrolases [64]. These ubiquitin-related factors may represent an initial line of defense against viral replication in enterocytes or they may be part of a viral strategy to elude mosquito immune responses [65–67]. Also, laminin, a component of the basal lamina that interacts with invading pathogens [68], was significantly upregulated in infected midguts. Among classical immune responses, members of the scavenger receptor family, leucine-rich receptors, and STAT factors were activated by viral infection in the midgut. Finally, cadherins, which act as binding proteins for DENV in mosquito cells, may serve as critical receptors during viral infection in mosquito cells [69].

**Fig 4:**
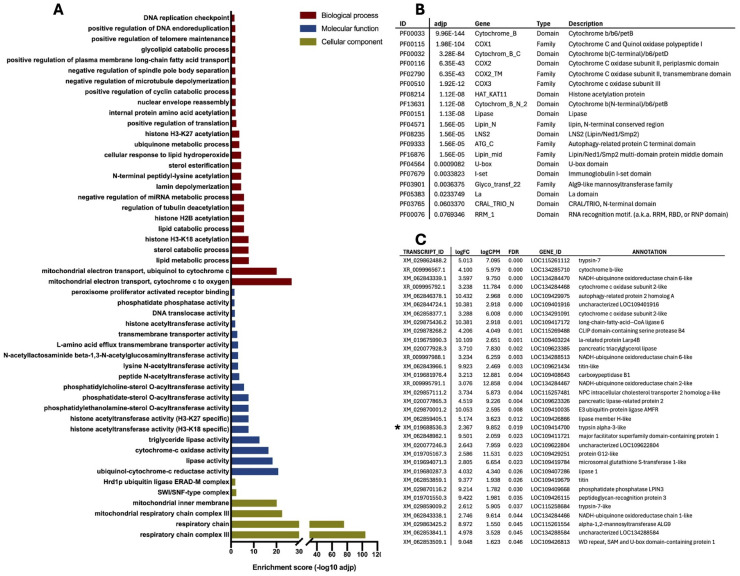
Selection of enriched terms in control midguts at 1 dpi, i.e., downregulated in response to CHIKV infection. **(A)** List of enriched Gene Ontology (GO) terms according to the three categories, Biological process (BP, red bars), Molecular Function (MF, blue bars) and Cellular Component (CC, green bars); **(B)** list of enriched Pfam domains, families and repeats: Pfam IDs and descriptions are reported as the adjusted p value (adjp); **(C)** list of differentially expressed (DE) contigs: transcript IDs, according to AalbF5 genome assembly, are reported with relative annotation; enrichment parameters are logFC (Fold Change) logCPM (Counts Per Million) and FDR, False Discovery Rate. The asterisk indicates XM_019688536.3, whose expression is analysed through RT-qPCR validation (Fig 9).

Several GO terms downregulated by the virus (i.e., upregulated in control midguts, Fig 4) were associated with lipid and lipoprotein metabolism, a pathway already known to be affected by arboviral infection of mosquitoes [70]. Other digestive processes also appeared to be modulated or suppressed by CHIKV, including triglyceride lipase activity and trypsin/carboxypeptidase activity. Among contigs overexpressed in control midguts were many peptidases, particularly carboxypeptidases, suggesting that blood meal digestion in infected midguts may be impaired to support viral survival and maintenance. The presence of DE contigs (Fig 4C) encoding lipase and peritrophin aligns well with findings from GO and Pfam enrichment analyses. One of the mosquito defense mechanisms against viral infections may involve targeting factors and pathways required for viral invasion of midgut cells through endosome formation and maturation [71]. Accordingly, an enrichment of GO and Pfam terms related to phagocytosis, recognition, vacuolar acidification, and endosomal vesicle fusion was observed in uninfected midguts (Fig 4A and 4B). Pathogen entry via clathrin-mediated endocytosis, which relies on receptors associated with clathrin, is another critical pathway [72]. This process requires specific chemical and physiological conditions, such as a pH below 6 and a particular lipid composition. Cholesterol and a pH below 6–6.5 are essential for the fusion of the alphavirus envelope with endosomes. Additional GO and Pfam terms more abundant in control midguts compared to infected midguts are related to the peritrophic matrix, chitin binding, and chitin metabolic processes. A specific repression of E3 ubiquitin ligases occurs in infected midguts, indicating that ubiquitination and proteasome formation may be altered by both the virus and the mosquito immune responses, as mentioned above. Recent studies have shown that the downregulation of genes involved in N-glycosylation, such as Alg9 and Alg3, is associated with the activation of the JNK pathway as a stress response [73]. Significant transcriptional changes in genes involved in the mitochondrial respiratory chain were also observed: while DENV and ZIKV infections were previously reported to affect mitochondrial metabolism, the transcriptional modulation of mitochondria-linked targets in CHIKV infected cells is less understood [74,75]. Furthermore, viral infection also appeared to affect factors involved in epigenetic modifications, which is not surprising since DNA/RNA methylation and histone acetylation (e.g., histone H2-H3 acetylation) have been previously associated to regulation of insect immune responses to pathogens [76,77].

### Gene expression profiles in carcasses at day 5 post infection

After midgut invasion, between 2 and 6 days post-infection, viral particles escape from the midgut to disseminate into the hemocoel and a general systemic transcriptional modulation of mosquito genes occurs in several mosquito organs [59,78]. To specifically focus on the systemic immune responses of mosquito organs, such as the fat body, and immune cells such as hemocytes, we dissected and removed the midguts from mosquitoes 5 days after an infectious or non-infectious (control) blood meal (Fig 1). RNA-seq datasets from infected and uninfected midguts and carcasses (whole females after midgut removal) were analyzed to evaluate CHIKV-induced transcriptional modulation.

**Fig 5:**
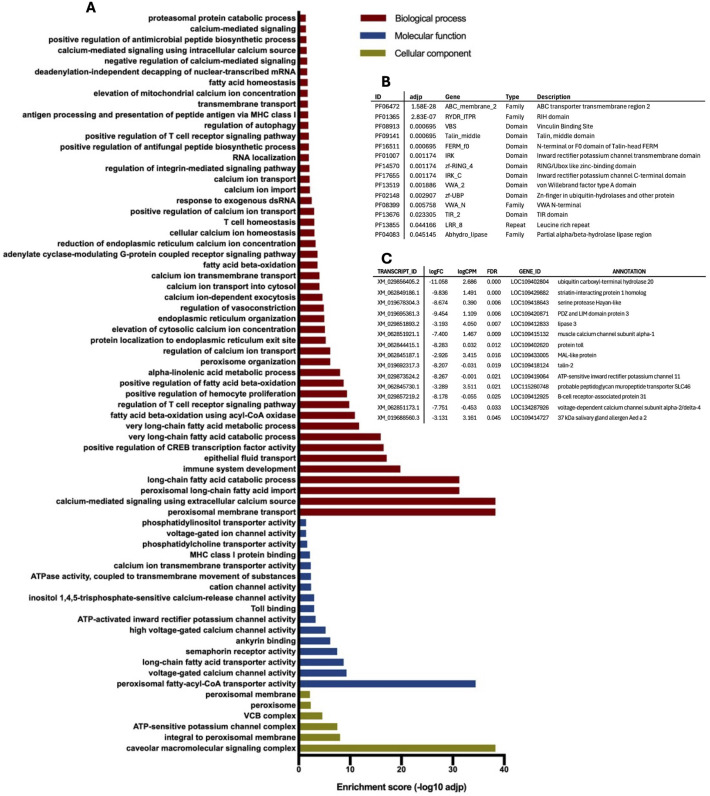
Selection of enriched terms in infected carcasses at 5 dpi, indicating upregulation in response to CHIKV infection. **(A)** List of enriched Gene Ontology (GO) terms according to the three categories, Biological process (BP, red bars), Molecular Function (MF, blue bars) and Cellular Component (CC, green bars); **(B)** list of enriched Pfam domains, families and repeats: Pfam IDs and descriptions are reported as the adjusted p value (adjp); **(C)** list of differentially expressed (DE) contigs: transcript IDs, according to AalbF5 genome assembly, are reported with relative annotation; enrichment parameters are logFC (Fold Change) logCPM (Counts Per Million) and FDR, False Discovery Rate.

Mosquito carcasses exhibited upregulated GO terms associated with peroxisomal organization and metabolism [79,80]. Key immune-related biological processes, such as calcium homeostasis, positive regulation of antimicrobial peptides, response to exogenous dsRNA, hemocyte proliferation and immune system development were also enriched in infected carcasses. Among enriched GO or Pfam terms and differentially expressed (DE) transcripts were also several processes related to lipid metabolism and fatty acid homeostasis (Fig 5). Also, the ubiquitin carboxyl-terminal hydrolase 20, a deubiquitinating enzyme involved in autophagy and cellular antiviral responses was strongly upregulated in infected carcasses compared to controls, and immunoglobulin domains —key components of immune-reactive molecules in invertebrates— were significantly upregulated upon pathogen challenge [81]. The 37 kDa salivary gland allergen Aed a 2 is highly similar (84% identity) to the juvenile hormone-binding protein (mJHBP) found in *Ae. aegypti*, a mosquito-specific protein that binds juvenile hormone III and is abundant in pupae and adults [82]. Disrupting the mJHBP gene using CRISPR-Cas9 led to impaired immune responses, including delayed antimicrobial peptide production, reduced immune gene expression, defective phagocytosis, and higher vulnerability to bacterial infections like *Serratia marcescens*. Additionally, mutant mosquitoes showed altered hemocyte populations and decreased hemocyte phagocytic activity [83]. Members of ABC transporter family, which are involved in translocation of various molecules across biological membranes, were highly enriched in CHIKV-infected *Ae. albopictus* carcasses suggesting their involvement in antiviral immunity. Among DEG, Talin, an integrin-like transmembrane receptor, may mediate immune responses in insect hemocyte-like cells [84,85]. Finally, a leucine-rich repeat-containing protein and a toll-like receptor, both upregulated in infected carcasses, are potentially involved in the defense pathways described above.

**Fig 6:**
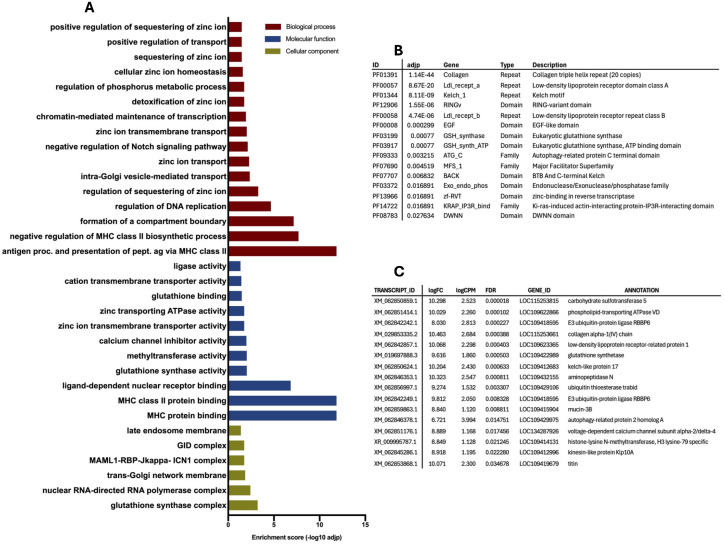
Selection of enriched terms in control carcasses at 5 dpi, indicating downregulation in response to CHIKV infection. **(A)** List of enriched Gene Ontology (GO) terms according to the three categories, Biological process (BP, red bars), Molecular Function (MF, blue bars) and Cellular Component (CC, green bars); **(B)** list of enriched Pfam domains, families and repeats: Pfam IDs and descriptions are reported as the adjusted p value (adjp); **(C)** list of differentially expressed (DE) contigs: transcript IDs, according to AalbF5 genome assembly, are reported with relative annotation; enrichment parameters are logFC (Fold Change) logCPM (Counts Per Million) and FDR, False Discovery Rate.

Among GO terms significantly downregulated during viral dissemination were factors involved in the ubiquitin pathway as the GID complex, which is associated with E3 ligase assembly and may play a role in the ubiquitination process [64]. Moreover, several protein families and domains, including RING domain-containing proteins, E3 ubiquitin protein ligases, and autophagy-related domains, were also downregulated by the presence of the virus (Fig 6). Certain Major Facilitator Superfamily (MFS) proteins play critical roles in immune processes, including viral invasion and pathogen resistance [86]. Additionally, a mucin protein in *Ae. aegypti* has been shown to interact with DENV, influencing viral infection dynamics [87]. Among other terms differentially expressed in carcasses at day 5, an LDL receptor was found downregulated in infected carcasses: notably, a reduction in LDL levels is linked to severe viral infections in *Ae. aegypti* mosquitoes infected with DENV [88]. Overall, the involvement of fatty acid and lipid metabolism in viral infection development has been previously discussed and plays a key role in the viral dissemination in the hemocoel.

### Distinct temporal and spatial immune responses: local and systemic alternatives

To get a wider overview on the modulation of immune-related pathways and gene families at local and systemic level following CHIKV infection, we first compiled a comprehensive list of *Ae. albopictus* immune-related gene families and then analyzed the transcriptional patterns governing mosquito immune responses to arboviral infection across different time points (T1 and T5) and body compartments (i.e., at 1 dpi in the midgut and at 5 dpi in the carcasses/hemolymph). To this end, immune-related genes from *Aedes* mosquitoes identified and/or catalogued in previous studies [48,70,89,90] were used to identify *Ae. albopictus* orthologs by tblastn searches and then further classified/refined using available annotations [91]. In addition, putative novel immune-related contigs were identified by searching specific annotation terms in our transcriptome. This way we obtained a final list of *Ae. albopictus* immune-related genes that included transcript isoforms with FPKM values ≥ 1 in at least one sample. The presence of multiple isoforms may result from differences in transcript length at the 5′ and/or 3′ UTRs, alternative splicing events, or occasional errors in transcript annotation. This becomes particularly relevant when truncated or spliced variants encode distinct domains with potential functional implications. In cases where discrepancies are observed, gene-specific analyses are required before drawing conclusions. Nonetheless, for most of the genes analyzed, both the expression profiles and the absolute FPKM values of the different isoforms exhibited a high degree of consistency. This catalogue is composed of 891 contigs grouped into the following immune-related families: ubiquitination, RNA interference, IMD, JAK-STAT, TOLL pathways, PGRP, scavenger receptors, LRR receptors, AMPs, PPO, CTL, CLIP, TEP, serpin, autophagy, and apoptosis (Supplementary S5 Data).

**Fig 7:**
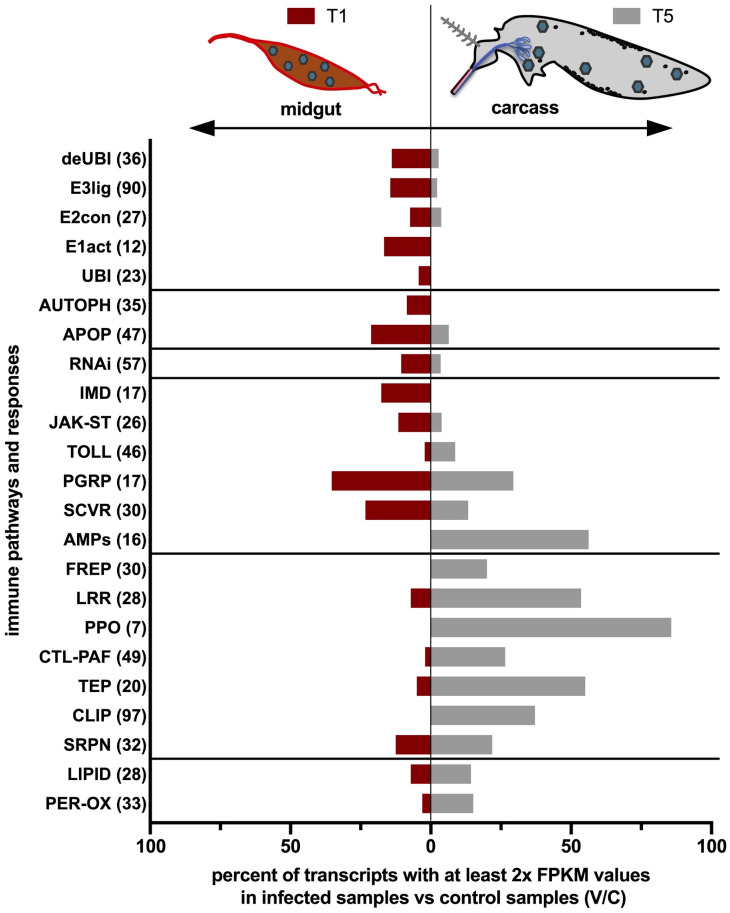
Transcriptional modulation of immune gene family members following viral challenge. The mirrored bar chart displays on the x-axis the percentage of transcripts that were at least twice as abundant in infected versus control samples from each immune group as listed on the y-axis (with the total number of family members in brackets). Red bars refer to data from midguts at 1 dpi, while grey bars represent data from carcasses at 5 dpi.

Variations of transcript abundance were evaluated comparing FPKM values of infected samples (V) versus control samples (C) and taking into consideration only transcripts with log2-transformed V/C ratio ≥ 1 or ≤ -1, that is at least twice as abundant in infected versus control samples, or viceversa (Supplementary S5 Data and Supplementary S1 Text). The mirrored bar graph in Fig 7 illustrates the percentage of contigs, within each immune group, with at least 2x FPKM values in infected samples compared to control samples in the midgut at T1 and in the carcass/hemolymph at T5. These variations in transcript abundance provide an overview of local and systemic *Ae. albopictus* immune defense responses to CHIKV infection offering the opportunity to focus on gene family and pathway levels rather than on individual genes.

**Fig 8:**
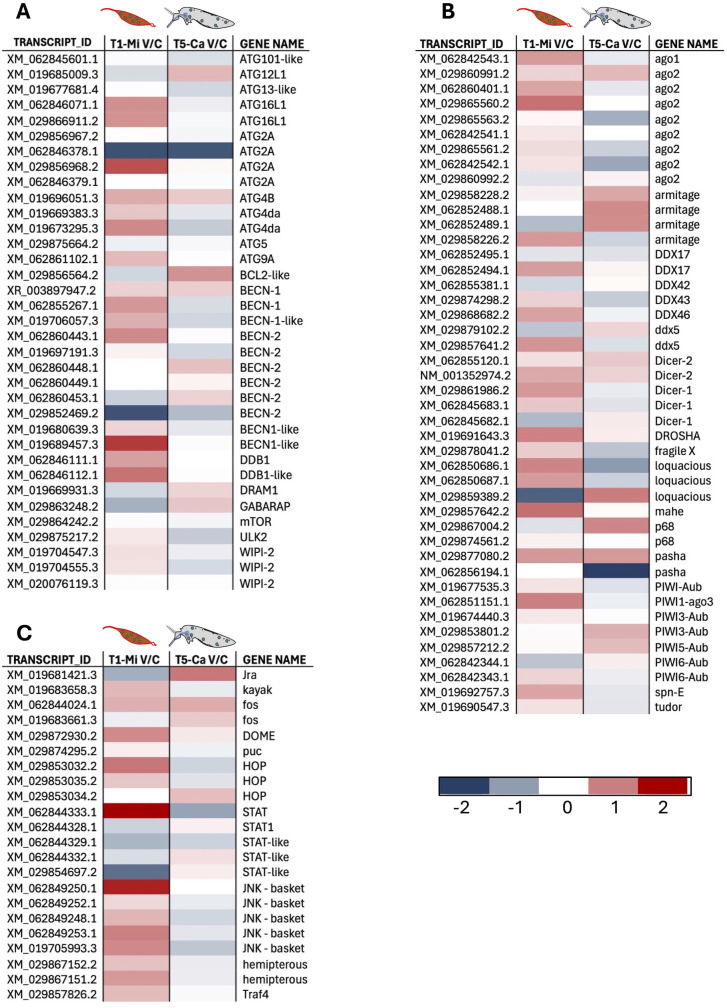
Heatmaps illustrating the transcriptional profiles of members of the (A) Autophagy, (B) RNAi and (C) JAK/STAT families. Each heatmap includes: the identifier of each contig (including possible isoforms) in the TRANCRIPT_ID column; the log2 values of the ratio between the FPKM values (+1 FPKM) of infected samples (V) and control samples (C), presented for midgut samples at 1 dpi (T1-Mi V/C) and carcass samples at 5 dpi (T5-Ca V/C), with a corresponding color legend below; the gene description or annotation provided in the GENE_NAME column.

Several immune gene families and pathways exhibited an increased transcriptional activity in the infected midgut at 1 dpi. RNA interference (RNAi) is recognized as a key antiviral mechanism in invertebrates and known to be involved in the response of *Aedes* mosquitoes to viruses such as DENV, ZIKV, and CHIKV [48,92] although significant upregulation of RNAi transcripts during the early stages of midgut infection was not consistently reported [46,78,93]. We observed upregulation of the RNAi pathway at 1 dpi in infected midguts, where it likely contributes to restricting early viral replication [94], and also found a limited, yet detectable, activation in infected mosquito carcasses at 5 dpi (Figs 7 and 8B). The evolutionarily conserved Toll, Imd, and JAK-STAT pathways appeared to play a role in limiting arbovirus replication, with the transcriptional activation of Imd and JAK-STAT components being especially evident in midguts at 1 day post-infection (dpi), whereas transcriptional upregulation of Toll family members was more pronounced in carcasses at 5 dpi (Figs 7 and 8C). It is important to note that within each family, both activators and repressors may be either up- or downregulated. Thus, their transcriptional modulation in response to viral challenge provides only a broad overview of the host response. A more comprehensive understanding of the underlying biological processes and functional implications will require further investigation at the level of individual genes and specific pathways.

**Fig 9:**
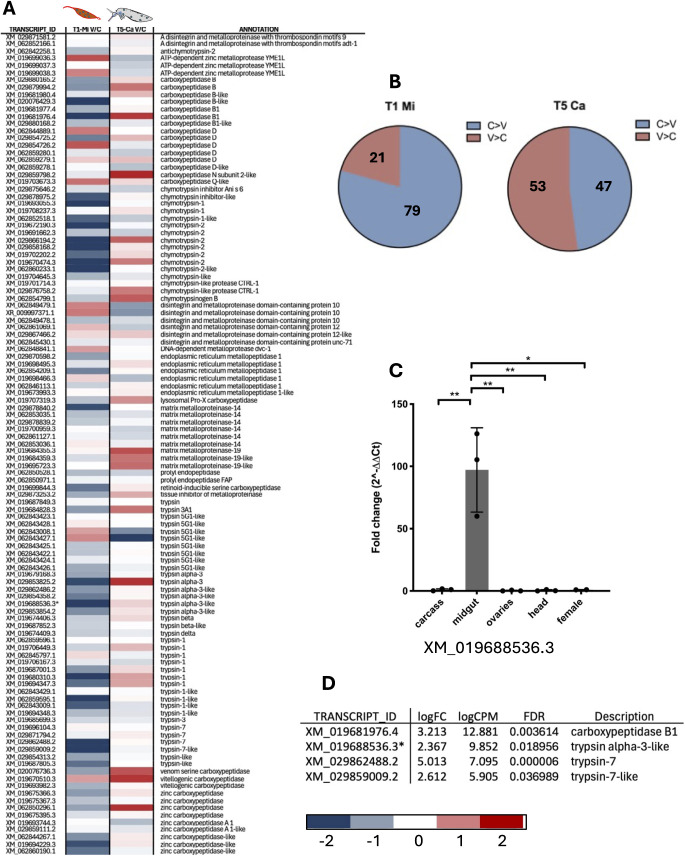
Transcriptional Modulation of Digestive Factors Upon CHIKV Infection. **(A)** Heatmap displaying transcriptional changes. **(B)** Pie chart illustrating the percentage of genes with a control/infected ratio >1 (blue) or <1 (red). **(C)** Validation of the trypsin gene XM_019688536.3 by RT-qPCR across different tissues, including whole females, dissected heads, ovaries, midguts, and carcasses (whole body excluding head, ovaries, and midgut). Statistical analysis was conducted using ordinary one-way ANOVA followed by Tukey’s multiple comparison test. **(D)** Summary of differentially expressed (DE) contigs within the digestive factor list.

**Fig 10:**
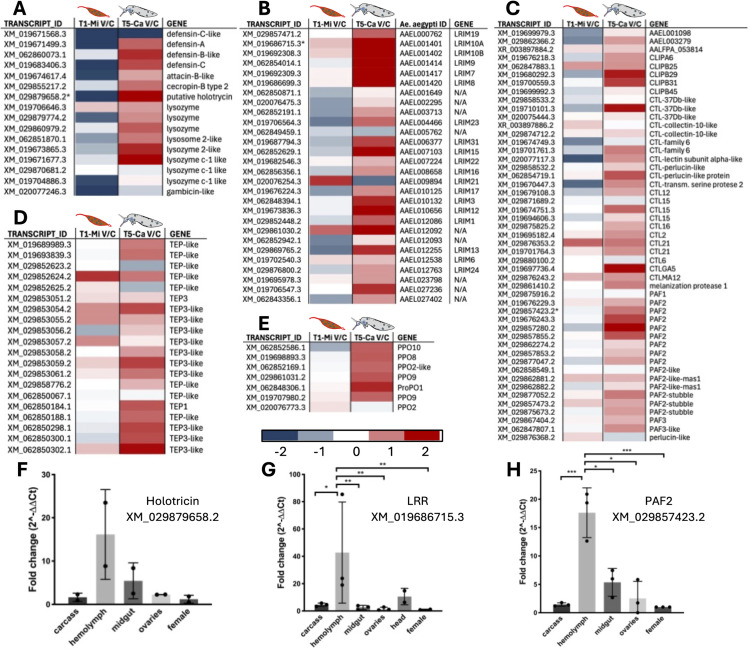
Heatmaps of Transcriptional Profiles Across Immune Categories. **(A)** AMPs (Antimicrobial Peptides); **(B)** LRR (Leucine-Rich Repeat proteins); **(C)** CTL-PAFs (C-type Lectins and Pathogen-Associated Factors); **(D)** TEP (Thioester-Containing Proteins); **(E)** PPO (Prophenoloxidase). Validation by RT-qPCR (contigs marked with asterisk): **(F)** Holotricin gene (XM_029879658.2); **(G)** LRR gene (XM_019686715.3); H. PAF2 gene (XM_029857423.2). RT-qPCR was performed across different tissues, including whole females, dissected heads, ovaries, midguts, and carcasses (whole body excluding head, ovaries, and midgut). Statistical analysis was conducted using ordinary one-way ANOVA followed by Tukey’s multiple comparison test.

Members of gene families involved in molecular and cellular homeostasis and turnover exhibited higher transcriptional abundance in infected versus uninfected midguts at T1 and in comparison, to carcasses at T5 (Fig 7). Among them, the upregulation of ubiquitination-related components resulted evident. This observation appears particularly relevant considering that post-translational protein modification regulates numerous biological processes [66], including immune responses of insects infected by various pathogens [67,95,96]. Additionally, growing evidence suggests that microbial pathogens may exploit the ubiquitin pathway to evade the host immune system [95]. Several genes appeared instead downregulated in the midgut at T1 in response to viral infection. Remarkably, significant downregulation of transcripts encoding digestive enzymes was observed in the early stages of CHIKV replication, when the virus crosses the epithelial barrier in the mosquito midgut. Searching our transcriptome using the keywords “trypsin”, “peptidase”, “carboxypeptidase”, “metalloprotease”, and setting as cutoff for inclusion an expression level ≥ 1 FPKM in at least one sample, we selected a total of 117 contigs. Among these, 79% were more abundant in control than in infected midguts, an unbalanced pattern that was not observed in carcasses at 5 dpi (Fig 9A and 9B). This downregulation of trypsins and other digestive enzymes may be induced by the virus and favor the infection as previously shown in DENV-infected *Ae. aegypti* [97]. Members of the AMPs, FREP and LRR families were also notably downregulated in midguts at 1 dpi, whereas they were clearly upregulated in carcasses at 5 dpi as discussed in more detail below (Fig C in S1 Text, Fig 10A and 10B).

As previously discussed, comparison of infected and uninfected transcriptional profiles at 5 dpi, corresponding to the viral diffusion stage in the mosquito hemolymph, revealed a limited but specific list of both immune and non-immune DEGs (Figs 5 and 6). The expression analysis of immune gene families’ members in carcasses at 5 dpi revealed a specialized systemic response, allowing for the identification of specific trends with transcriptional activation of immune-related gene families in infected carcasses as compared to uninfected controls (Figs 7 and 10). Mosquito hemocytes, the only immune cells capable of mounting both humoral and cellular immune responses, play a central role in defense against bacteria, fungi, and parasites. However, their involvement in antiviral responses is not yet fully understood [98]. The classic antiviral TOLL immune pathway appears to be activated in the hemolymph but to a lesser extent than in the early midgut stage (Fig 7). Notably, several pattern recognition receptors (PRRs) were upregulated in the hemolymph; more specifically, members of the FREP, LRR, CTL and TEP families were transcriptionally activated in infected carcasses at 5 dpi both in comparison to uninfected carcasses and in comparison to infected midguts at 1 dpi. Thioester-containing protein (TEP) family members play a key role in antibacterial and anti-plasmodium defense and lectins, which can recognize carbohydrate components of viral envelopes, promote opsonization activating immune mechanisms such as the PPOs (Phenoloxidases) cascade and phagocytosis. Melanization is indeed another hemolymph-specific immune process and, accordingly, we found that several members of the PPOs, CLIP proteases and PAF (Phenoloxidase-activating factors) families were more abundant in infected carcasses than in uninfected controls. Antimicrobial peptides (AMPs) were also found abundant in the hemolymph and specifically activated at this time point. Also, in addition to classical AMPs known to be activated by viral infection, we observed differentially expressed (DE) genes encoding glycine-rich peptides and a putative holotricin, which may represent a novel AMP involved in the immune response to invading pathogens [99–101]. To verify the tissue-specific expression profiles of a few selected candidate genes (marked with asterisks in the heat maps of Figs 9 and 10), we dissected *Ae. albopictus* female mosquitoes and isolated hemocytes using the proboscis clipping technique. Notably, two of the analyzed candidates—a member of the Leucine-Rich Repeat (LRR) protein family and a Pro-phenoloxidase Activating Factor 2 (PAF2)—were found to be specifically expressed in circulating hemocytes. Additionally, the putative AMP holotricin displayed a highly enriched expression profile in the hemocytes (Fig 10F–10H).

## Discussion

In this study we examined the transcriptional responses of *Ae. albopictus* to chikungunya virus infection focusing our attention specifically on midguts at one dpi and on carcasses (mosquito body after midgut removal) at five dpi. These two tissue/organs and timepoints were chosen because they identify two crucial stages of the viral lifecycle and of the mosquito immune responses: (i) the early phase, taking place after viral ingestion with the blood meal and involving invasion and replication within midgut epithelial cells; (ii) the late phase, when viral particles are disseminated through the hemolymph and replicate in various tissues before reaching salivary glands for transmission. This experimental scheme was expected to provide further insights into *Ae. albopictus*-CHIKV interactions, since previous RNA-seq studies were restricted to the analysis of immune responses in the midgut at 1–2 dpi and in the head/thorax at 8 dpi [46–48,102]. It should be emphasized that our study could take advantage of the recently released AalbF5 genome assembly, yielding a reference transcriptome of 38,020 contigs. A comparison between our DEG dataset and those from previous transcriptomic studies [48] is provided in Supplementary S1 Text (Supplementary S6 Data, Fig D and Fig E in S1 Text). To gain a more detailed understanding of the dynamics involved in mosquito defense against the invading CHIK virus, a comprehensive list of 891 *Ae. albopictus* immune-related genes/contigs was compiled, and their expression profiles in infected versus control samples was evaluated. These genes were grouped into different functional immune categories, which included ubiquitination, RNA interference (RNAi), Toll/IMD/JAK-STAT pathways, scavenger receptors, leucine-rich repeat (LRR) proteins (involved in pathogen recognition), antimicrobial peptides (AMPs), prophenoloxidase (PPO) pathways (involved in melanization and immune priming), autophagy and apoptosis. These transcriptomic profiles provided key insights into how mosquito immune defenses shift over time and across different organs to combat CHIKV infection (Fig 7), revealing distinct immune responses of local (midgut at 1 dpi) and systemic immunity (hemolymph at 5 dpi). Notably, there was minimal overlap between differentially expressed genes (DEGs) at 1 and 5 dpi in the midgut and at 5 dpi in the carcasses, supporting the idea that the mosquito immune system operates independently in these two distinct phases. A similar finding was previously reported in DENV-infected *Ae. aegypti* [78], with differential transcript modulation in response to infection observed in the midgut, carcasses, and salivary glands.

During the initial response the virus faces multiple barriers, including gut immunity, interactions with the microbiota, digestive enzymes, and formation of the peritrophic matrix. Even though the involvement of the RNAi pathway in mosquito immune defense against arboviruses was recently found more complex than originally assumed [94,103], in our study RNA interference appeared to play a pivotal role in antiviral defense during the early stage of CHIKV infection. This was clearly indicated by DE, Pfam, and GO analyses, which showed a significant upregulation in infected midguts of RNA helicases and of components of both the PAN2-PAN3 deadenylase complex and of the RNAi pathway [94,103–106]. The early immune response also involved ubiquitination, as suggested by the upregulation in infected midguts of E3 ubiquitin ligases and hydrolases, which regulate early antiviral activity. Ubiquitination targets proteins for degradation by addition of ubiquitin and it is counteracted by deubiquitination, which removes or modifies ubiquitin residues by different mechanisms [67]. Beyond its role in cellular homeostasis and other biological pathways, ubiquitination also plays a regulatory role in immune pathways, including the Toll, Janus kinase (JAK)/signal transducer and activator of transcription (STAT), and immune deficiency (IMD) pathways, during immune responses to bacterial, viral, and fungal infections in insects [66,107]. A direct involvement of the ubiquitination pathway in viral transmission by infected mosquitoes was already demonstrated in DENV-infected *Ae. aegypti* [96]. Accordingly, in our transcriptome, we observed a widespread upregulation of ubiquitination-related enzymes and factors, particularly during the early stages of viral invasion in the midgut. This response may result from immune defense mechanisms activated by the mosquito or from metabolic alterations induced by the virus or, perhaps, by a combination of both processes. Transcripts associated with autophagy and apoptosis were also more abundant in infected midguts compared to controls, confirming that viral and/or cellular proteins may be targeted for degradation, and that apoptosis may either serve as a defense mechanism or be triggered by the infection.

Metabolic shifts caused by CHIKV infection were also evident in the midgut at one dpi. For instance, laminin, a basal lamina component, was upregulated during early invasion of the midgut, possibly influencing immune regulation through complement factor LRIM1 [68,108]. Indeed, laminin downregulation was previously reported to reduce malaria oocyst intensity and enhance phagocytosis in *in vivo* and cell-based RNAi assays [68,108]. Moreover, laminin was also suggested to regulate the expression of the complement factor LRIM1 during immune challenges in *Anopheles* mosquitoes [68]. Chitin metabolism and peritrophic matrix-associated genes were also affected, suggesting that changes in barrier function could influence viral entry. Infected midguts exhibited a significant downregulation of genes associated with lipid metabolism and digestion, including trypsins and carboxypeptidases. Indeed, a reduction in a lipoprotein receptor has been previously observed in *Ae. aegypti* mosquitoes following DENV infection and suggested to be linked to the viral invasion pathway via endocytosis [109]. Downregulation of lipase, an enzyme involved in lipid digestion in the mosquito midgut, may represent a viral strategy to mitigate potential damage [110]. Lipin, another factor downregulated by the virus, also plays a role in reproductive metabolism [111]. Alteration of peptidase expression could impact both blood digestion and viral infection dynamics, potentially facilitating viral replication by modulating mosquito physiology. We analyzed the tissue-specific expression profile of the differentially expressed contig XM_019688536.3, encoding for a putative trypsin-like, which was downregulated in midguts at 1 dpi. Notably, we confirmed its specific expression in the midgut, and the significant reduction in transcript abundance upon viral infection suggests that the virus may regulate its expression to counteract its enzymatic activity. Among immune pathways, the IMD and JAK-STAT pathways were predominantly activated by the virus at 1 dpi in the midgut, whereas the Toll pathway appeared to play a greater role in the later response, particularly in carcasses at 5 dpi. The JAK/STAT pathway is well-known for its transcriptional activation upon viral infection and its downstream role in triggering effective immune responses [24]. Similarly, the enhanced transcription of pathogen recognition receptors in response to viral infection suggests that scavenger receptors and PGRPs may contribute to both early midgut immunity and later responses in the hemocoel. Scavenger receptors are a heterogeneous family of molecules known to recognize pathogen- and danger-associated molecular patterns (PAMPs and DAMPs) in insects [43]. In contrast, the upregulation of leucine-rich repeat (LRR) proteins, primarily in the carcasses at 5 dpi, indicates their likely involvement in immune signaling mediated by hemocytes and fat body cells in the hemolymph. Indeed, members of the leucine-rich protein family are implicated in pathogen immune recognition, including arboviruses, though their specific roles remain unclear [70,112]. These results are in agreement with previous studies on CHIKV- and DENV-infected *Ae. albopictus* where several components of Toll, IMD and JAK/STAT pathways resulted co-regulated during midgut early infection stages [48,113].

As the virus enters the hemocoel between 1 and 6–8 dpi, the mosquito immune system activates systemic defenses, primarily mediated by hemocytes and fat body cells. Numerous studies have explored the different pathways used by viruses to spread from the midgut to other organs. One such mechanism involves the initial replication of the virus within infected midgut cells, followed by its spread to the midgut tracheal system, which facilitates further viral replication and its subsequent release into the hemolymph. Once in circulation, the virus continues to infect and replicate in various tissues, including the fat body, hemocytes, and salivary glands, all of which contribute to sustain viral replication [98,114]. The analysis of carcasses at five dpi revealed the co-regulation of various gene family members. The higher abundance, upon viral infection, of transcripts encoding pattern recognition receptors (e.g., TEP, LRR, and FREP) indicates enhanced immune surveillance, as well as the involvement of cellular processes as melanization and phagocytosis through phenoloxidase activation and upregulation of specific receptors. We also found a significant upregulation of antimicrobial peptides, including a newly identified holotricin [101,115], in carcasses at 5 dpi. It is well known that hemocytes produce various soluble molecules, including antimicrobial peptides (AMPs), enzymes, and opsonins, to combat pathogens in the hemolymph. Tissue-specific expression analysis by RT-qPCR further clarified the expression patterns of some candidate genes within these families. For example, the contig XM_019686715.3, which encodes a member of the LRR protein family, was specifically expressed in circulating hemocytes (Fig 10G). Interestingly, this putative LRR protein is the ortholog of *Ae. aegypti* AAEL001401, which was found to be significantly upregulated in the Key West strain of *Ae. aegypti* three days after CHIKV ingestion [70,116]. This concordance suggests a conserved role for this receptor in the innate immune response of *Aedes* mosquitoes against invading viruses. Prophenoloxidase (PPO), exclusively produced by hemocytes, plays a key role in pathogen defense, coagulation, cuticle hardening, and pigmentation [98]. The role of melanization in mosquito innate immunity has been primarily demonstrated as a defense mechanism against invading bacteria, fungi, and parasites [14,15,60]. However, PPO upregulation and activation have also been observed in *Ae. aegypti*, *Armigeres subalbatus*, and *Lymantria dispar* upon infection with different viruses (reviewed in [98]). Moreover, knockdown or inhibition of PPO led to increased viral load and mortality, pointing at a relevant role in antiviral immunity, perhaps by melanizing infected cells or recognizing viral glycoproteins through lectins, although the exact mechanism remains unclear and may vary depending on the virus and insect species. We observed a coordinated transcriptional upregulation of members of the melanization pathway such as C-type lectins, CLIP serine proteases, prophenoloxidase, and prophenoloxidase-activating factors in infected carcasses at 5 dpi compared to controls. Additionally, the contig XM_029857423.2, which encodes PAF2, appeared to be tissue-specifically expressed in circulating hemocytes.

Beyond melanization, an additional important arm of insect immune defenses is represented by antimicrobial peptides. AMPs are highly conserved immune effectors found in all living organisms, with insects exhibiting a remarkable diversity and abundance. In insects, AMPs are primarily produced by fat bodies and hemocytes and display broad activity against various pathogens, including viruses [14,98]. DENV infection is known to lead to overexpression of AMPs such as defensins, cecropins, gambicin, diptericin, and attacin in *Ae. aegypti* [117] and cecropin and defensin knockdown results in increased viral load, suggesting their antiviral role [118]. While direct evidence of AMPs functioning as an antiviral defense in mosquito hemocytes is lacking, these cells express several key AMPs. In line with these observations, we found an upregulation of transcripts encoding *Ae. albopictus* cecropin, defensins, attacin, and lysozymes in infected carcasses at 5 dpi; conversely, AMP transcript abundance was reduced at 1 dpi in infected midguts compared to controls (Fig 10A). Among AMPs, we found upregulation at 5 dpi in infected carcasses of XM_029879658.2, a contig which encodes a putative holotricin. Originally identified in *Holotrichia diomphalia* larvae [115], this antimicrobial peptide has since been found in other insects [101], including *Ae. aegypti* [78], where it is highly expressed in carcasses under baseline conditions [100]. However, its transcriptional response to DENV infection varies, showing downregulation in carcasses at 1 dpi and upregulation at 4 dpi [78], a pattern similar to the one we observed here. Tissue-specific expression analysis of this putative *Ae. albopictus* holotricin (XM_029879658.2) confirmed its predominant expression in circulating hemocytes, with lower expression levels detected in the midgut.

GO enrichment analysis of infected carcasses further revealed the enrichment of genes linked to lipid metabolism, peroxisomal function, and overall immune system activation. In *Ae. aegypti*, fatty acid metabolism has been shown to play a critical role in the immune responses to DENV infection [119] and fat body cells serve as the primary site for lipid metabolism [120]. In *An. gambiae* a lipocalin family member mediates innate immune priming in response to *Plasmodium* infections [121], a mechanism that likely enhances the mosquito ability to respond to subsequent infections. ABC transporters, which are known to contribute to pathogen defense in *Ae. aegypti* [122], were also upregulated. Involvement of members of this family in antiviral immunity is supported by the observation that RNAi-mediated silencing of an ABC transporter in *Drosophila* increases susceptibility to viral infections [123]. Additionally, certain ABC subfamily transporters act as antagonists of *Plasmodium* infection in *An. gambiae* [124]. Interestingly, ubiquitination-related pathways were significantly suppressed at this stage, indicating potential viral interference with protein degradation mechanisms, which may act as an immune evasion strategy. Additionally, disruptions in zinc metabolism and fatty acid homeostasis were observed, possibly affecting mosquito physiology and vector competence. Zinc (Zn) metabolism is essential for the growth, development, and immune function of both insects and microbial pathogens. Depending on its concentration and method of application, Zn can have either positive or negative effects on these organisms. Insects produce Zn-binding proteins and transporters to regulate Zn levels and restrict their availability to invading pathogens [125,126]. It has been shown that, in addition to members of the LDLR (Low-Density Lipoprotein Receptor) and CTL families, highly glycosylated heparan sulfate proteins and laminin receptors can also function as attachment factors in mosquitoes. These molecules enhance viral particle capture by cells without facilitating entry, while co-receptors or specific entry receptors are required for viral internalization [127].

Overall, our study confirms and extends previous findings on *Ae. albopictus*-CHIKV interactions, highlighting the dynamic nature of the mosquito immune response, with localized midgut defenses activated during the early phase of infection, followed by systemic immune responses in the hemolymph as the virus disseminates. Notably, Modahl et al. identified 1,793 DEGs in *A. albopictus* midguts at 1 dpi with CHIKV. When compared with the coherent set of 145 DEGs presented here, a meaningful overlap emerged, encompassing several genes and domains of functional relevance, including E3-ubiquitin ligase, DE-cadherin, peroxisomal oxidase, scavenger receptors, and digestive enzymes (Supplementary S1 Text). The recurrence of these targets across independent datasets strengthens the view that key molecular pathways are consistently mobilized during the early midgut response to CHIKV infection. Moreover, data reported here clearly show how CHIKV infection affects several immune and metabolic pathways to establish persistence in the mosquito and provide valuable insights into vector competence and potential targets for mosquito-based viral control strategies.

## Supporting information

S1 Text This file contains the following information: Experimental infections (supporting method); Evaluation of infection rates and intensities by RT-qPCR (supporting result); DE analysis in Midguts at T5 (supporting result); DE groups’ comparison (supporting result); Downregulation of immune families upon viral challenge (supporting result); Primers’ sequences (supporting material); Comparison with previous transcriptomic datasets.
(DOCX)

S1 Data Transcriptome (38.020 contigs database). Legend: Annocript outputs: TRANSCRIPT_ID: ID from AalbF5 reference genome (GCF_035046485.1, NCBI) dataset; ANNOTATION: gene description; GENE_ID: LOCxx ID from previous assembly; TYPE: transcript variants, isoforms or single mRNA; FPKM values of each replicate (marked grey) and averaged values (marked yellow).; T1C_F1; T1C_F3; T1C_Fe; T1C_M1; T1C_M2; T1C_Mi; T1V_F1; T1V_F2; T1V_Fe; T1V_M1; T1V_M2; T1V_Mi; T5C_C1; T5C_C2; T5C_C3; T5C_Ca; T5C_F1; T5C_F2; T5C_F3; T5C_Fe; T5C_M1; T5C_M3; T5C_M4; T5C_Mi; T5V_C2; T5V_C3; T5V_Ca; T5V_F2; T5V_F3; T5V_Fe; T5V_M1; T5V_M2; T5V_M4; T5V_Mi.
(XLSB)

S2 Data Differential Expression Analysis (DE) as obtained by EdgeR. Column headers as follows: TRANSCRIPT_ID, ID as from the Rockefeller A5 dataset; sampleA and sampleB, samples compared; logFC, log2 fold change (log base 2 of the fold change in gene expression between the two experimental conditions); logCPM, log2 counts per million (log base 2 of the normalized expression levels for a given gene across all samples); Pvalue, probability that the observed difference in gene expression occurred by chance under the null hypothesis; FDR, false discovery rate (multiple testing corrected P value using the Benjamini-Hochberg method; when FDR ≤ 0.05, the gene is considered significantly differentially expressed after adjusting for multiple comparisons). FPKM, Fragment per Kilobase per Million Reads, of the different replicates are also reported. The different comparisons are reported in the different worksheets as indicated: T1 and T5, 1 dpi and 5 dpi; Mi_V and Mi_C, infected and uninfected midguts; Ca_V and Ca_C, infected and uninfected carcasses; UP, upregulated.
(XLSX)

S3 Data Pfam enrichment analysis. Legend: Annocript outputs: ID: PFAM ID code; counts_AalbF5: PFAM counts in the transcriptome; counts_DE: PFAM counts in the different DE groups as indicated in the different excel sheets; pval and adjp are statistical tests where adjp (adjusted P value) is the multiple testing corrected P value; Gene: gene containing the indicated Pfam; Type: Family, Domain, Coiled-coil, Repeat; Description: description of the Pfam.
(XLSX)

S4 Data GO terms enrichment analysis. Legend: ID: GO ID code; counts_AalbF5: GO counts in the transcriptome; counts_DE: GO counts in the different DE groups as indicated in the different excel sheets; pval and adjp are statistical tests where adjp (adjusted P value) is the multiple testing corrected P value; definition: description of the GO; division: GO division: P: Biological Process – e.g., immune response, metabolism; F: Molecular Function – e.g., ATP binding, enzyme activity; C: Cellular Component – e.g., nucleus, membrane.
(XLSX)

S5 Data Immune families. Legend: TRANSCRIPT_ID: contig ID code; DESCRIPTION: gene description as reported from annotation; GENE_ID: LOCxx ID code (AalbF5 annotation); T1_Mi_V-C and T5_Ca_V-C: log2-transformed values of the ratio between the FPKM values (+1 FPKM) of infected samples (V) and control samples (C) in midguts at 1 dpi and carcasses at 5 dpi; GENE_NAME: gene name, according to annotation and/or homology with known proteins; in the LRR sheet: AEGYPTI_QUERY: ID of *Ae. aegypti* genes used to search *Ae. albopictus* orthologs. Each sheet provides a detailed overview of key characteristics—including gene identifiers, functional annotations, and expression profiles—of immune-related genes belonging to the specified families: AMP, antimicrobial peptides; APOPTOSIS, genes involved in apoptosis; AUTOPHAGY, CLIP, FREP, IMD, JAK-STAT, LIPID METABOLISM, LRR, OX-PEROXIDASE, PGRP, PAF-CTL, PO-PPO, RNAi, SCVR, SRPN, TEP, TOLL-SPZ-REL, TRYPSINS, UBI, UBI_E1-ACT, UBI_E2-CONJ, UBI_E3-LIG, UBI_DE-UBI.
(XLSX)

S6 Data Databases comparison. List of differentially expressed genes (DEGs) simultaneously identified in both Modahl’s dataset and our dataset, including previous ID(s), gene ID, product description, Pfam ID, and Pfam description.
(XLSX)
