# Supplementary material for: Temporal and spatial profiling of Aedes albopictus immune responses to chikungunya virus infection
Source: PLoS Negl Trop Dis. 2025 Oct 3;19(10):e0013588. doi: 10.1371/journal.pntd.0013588 (PMC12510663; doi:10.1371/journal.pntd.0013588)
Supplement: S1 Text — This file contains the following information: Experimental infections (supporting method); Evaluation of infection rates and intensities by RT-qPCR (supporting result); DE analysis in Midguts at T5 (supporting result); DE groups’ comparison (supporting result); Downregulation of immune families upon viral challenge (supporting result); Primers’ sequences (supporting material); Comparison with previous transcriptomic datasets. (DOCX) [file pntd.0013588.s001.docx]

**Supporting information**

**Experimental infections**

Three infection experiments were independently carried out as detailed in the Table A (Ca: carcasses; Mi: midguts; Fe: females; CHIKV strain: CT665 Giambi; titer: 2.98x10^7^/ml).

**Table A. Infection experiments and sample collection.**

|  | **Replicate 1** | | | | **Replicate 2** | | | | **Replicate 3** | | | |
| --- | --- | --- | --- | --- | --- | --- | --- | --- | --- | --- | --- | --- |
|  | **1 dpf** | | **5 dpf** | | **1 dpf** | | **5 dpf** | | **1 dpf** | | **5 dpf** | |
|  | **C-T1** | **V-T1** | **C-T5** | **V-T5** | **C-T1** | **V-T1** | **C-T5** | **V-T5** | **C-T1** | **V-T1** | **C-T5** | **V-T5** |
| Mi | **20** | **20** | **20** | **20** | **20** | **21** | **21** | **20** | **20** | **20** | **20** | **20** |
| Ca | **10** | **10** | **10** | **10** | **10** | **10** | **11** | **10** | **10** | **13** | **10** | **10** |
| Fe | **6** | **6** | **5** | **5** | **5** | **5** | **5** | **5** | **8** | **5** | **5** | **5** |
| *RT-qPCR* | *-* | *-* | *-* | *13* | *-* | *-* | *-* | *15* | *-* | *-* | *-* | *15* |

Samples collected from infected (V) and uninfected mosquitoes. T1: 1 day post-infection; T5: 5 days post-infection. The underlined groups are those from which samples analyzed by RT-qPCR were selected.

Samples were collected and pools were preparated as follows:

T1 CHIKV (V-T1): midgut pools (20-21 Mi); carcass pools (10-13 Ca); female pools (5-6 Fe).

T5 CHIKV (V-T5): 20-21 individual Mi; 10 individual Ca; 5 individual Fe.

T1 Control (C-T1): midgut pools (20 Mi); carcass pools (10 Ca); female pools (5-8 Fe).

T5 Control (C-T5): 20-21 individual Mi; 10-11 individual Ca; 5 individual Fe.

**Evaluation of infection rates and intensities by RT-qPCR**

Real time PCR were performed to identify and quantify CHIKV in carcasses (CA) and females (FE) 5 days post infected blood meal from each infection experiments.

**First Replicate**

Real time PCR were performed on the following samples at 5 dpi (13 samples analysed by Real Time PCR: 10 CA, 3 FE).

| **Sample** | **CHIKV titer** |
| --- | --- |
| T5_V CA1 | 1.14x10^6^ PFU/ml |
| T5_V CA2 | 4.48x10^5^ PFU/ml |
| T5_V CA3 | 1.43x10^6^ PFU/ml |
| T5_V CA4 | 4.98x10^6^ PFU/ml |
| T5_V CA5 | 1.9x10^1^ PFU/ml |
| T5_V CA6 | 3.45x10^5^ PFU/ml |
| T5_V CA7 | 8.33x10^5^ PFU/ml |
| T5_V CA8 | 4.29x10^-1^ PFU/ml |
| T5_V CA9 | 5.05x10^5^ PFU/ml |
| T5_V CA10 | 1.13x10^6^ PFU/ml |
| T5_V FE1 | 1.91x10^-1^ PFU/ml |
| T5_V FE2 | 2.47x10^5^ PFU/ml |
| T5_V FE3 | - |

**Second replicate**

Real Time PCR were performed on the following samples at 5 dpi (15 samples analysed by Real Time PCR: 10 CA, 5 FE).

| **Sample** | **CHIKV titer** |
| --- | --- |
| T5_V CA1 | 6.51x10^1^ PFU/ml |
| T5_V CA2 | 0.58x10^1^ PFU/ml |
| T5_V CA3 | 2.96x10^1^ PFU/ml |
| T5_V CA4 | 3.56x10^6^ PFU/ml |
| T5_V CA5 | 6.91x10^6^ PFU/ml |
| T5_V CA6 | 9.40x10^2^ PFU/ml |
| T5_V CA7 | 2.41x10^6^ PFU/ml |
| T5_V CA8 | 3.84x10^1^ PFU/ml |
| T5_V CA11 | 5.31x10^6^ PFU/ml |
| T5_V CA12 | 3.43x10^6^ PFU/ml |
| T5_V FE6 | 1.79x10^7^ PFU/ml |
| T5_V FE7 | 1.80x10^3^ PFU/ml |
| T5_V FE8 | 4.39x10^7^ PFU/ml |
| T5_V FE9 | 6.29x10^7^ PFU/ml |
| T5_V FE10 | 1.73x10^7^ PFU/ml |

**Third replicate**

Real Time PCR were performed on the following samples at 5 dpi (15 samples analysed by Real Time PCR: 10 CA, 15 FE).

| **Sample** | **CHIKV titer** |
| --- | --- |
| T5_V CA1 | 8.96x10^6^ PFU/ml |
| T5_V CA2 | 9.50x10^2^ PFU/ml |
| T5_V CA3 | 4.37x10^7^ PFU/ml |
| T5_V CA4 | 2.99x10^6^ PFU/ml |
| T5_V CA5 | 1.2x10^5^ PFU/ml |
| T5_V CA6 | 6x10^6^ PFU/ml |
| T5_V CA7 | 2.16x10^6^ PFU/ml |
| T5_V CA8 | 4.39x10^6^ PFU/ml |
| T5_V CA9 | 2.97x10^6^ PFU/ml |
| T5_V CA10 | 1.67x10^7^ PFU/ml |
| T5_V FE1 | 6.70x10^7^ PFU/ml |
| T5_V FE2 | 1.35x10^7^ PFU/ml |
| T5_V FE3 | 6.81x10^7^ PFU/ml |
| T5_V FE4 | 1.14x10^7^ PFU/ml |
| T5_V FE5 | 2.20x10^7^ PFU/ml |

**DE analysis in Midguts at T5 (5 dpi)**

Results of DE analysis of CHIKV infected and control midgut at 5 dpi are reported in Table B, in Fig A and in S1 and S2 Data Excel Files.

**Table B. Summary of DE analysis in midguts at 5 dpi.**

|  | FDR<0.05 | |
| --- | --- | --- |
|  | CHIKV UP | Control UP |
| Mi T5 V vs C | 106 | 46 |


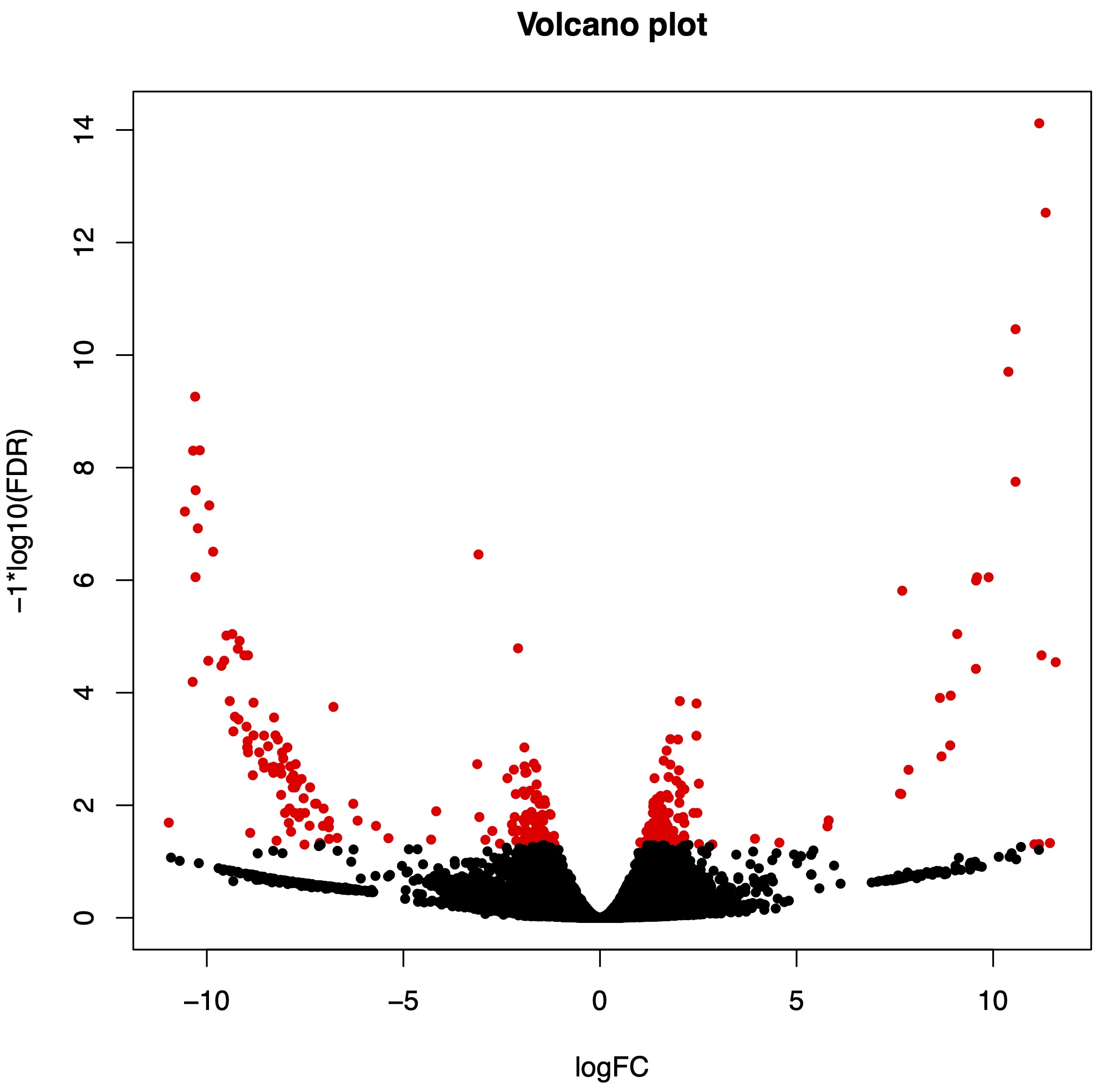


**Fig A. Volcano plots displaying the results of the differential expression (DE) analysis in midguts at TP5.** The x-axis represents Log2 fold change (FC), while the y-axis represents -Log10 false discovery rate (FDR). DE genes are highlighted in red. Genes differentially expressed in the control groups are shown on the right side of the graphs, whereas those in the infected groups are displayed on the left side.

**DE groups’ comparison**

Six differentially expressed (DE) gene groups were analysed and compared to identify commonalities and overlaps, providing insight into shared expression patterns, biological pathways or regulatory mechanisms (Fig B).


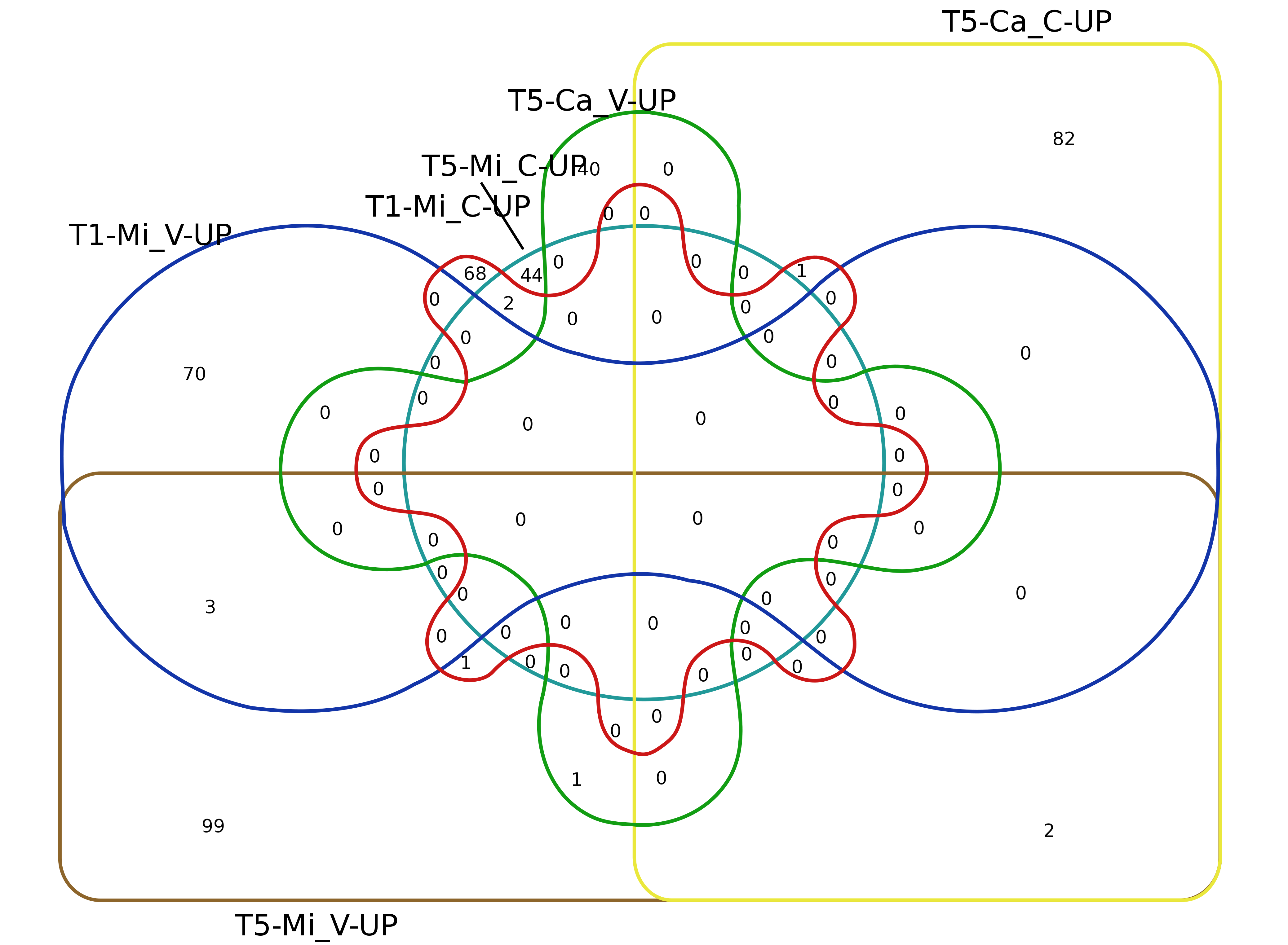


**Fig B. Venn diagram.** Venn diagram showing overlaps of DE genes from the 6 groups: T1-Mi, V-UP and C-UP; T5-Ca, V-UP and C-UP and T5-Mi, V-UP and C-UP.

Only 10 genes are shared in more than one group as detailed in the Table C. Venn diagram was obtained using the server: <https://bioinformatics.psb.ugent.be/webtools/Venn/>

**Table C. List of shared contigs.**

| GROUPS | N° CONTIGS | TRANSCRIPT_ID | ANNOTATION |
| --- | --- | --- | --- |
| T1-Mi_V-UP and T5-Mi_V-UP | 3 | XM_029865241.2 | FHIP family protein AAEL005291 |
|  |  | XM_062859701.1 | kinase D-interacting substrate of 220 kDa |
|  |  | XM_019685298.3 | major facilitator superfamily domain-containing protein 1 |
| T1-Mi_C-UP and T5-Ca_C-UP | 1 | XM_062846378.1 | autophagy-related protein 2 homolog A |
| T1-Mi_C-UP and T5-Mi_V-UP | 1 | XM_029870425.2 | uncharacterized LOC115265418 |
| T1-Mi_C-UP and T5-Mi_C-UP | 2 | XM_062848982.1 | major facilitator superfamily domain-containing protein 1 |
|  |  | XM_019698291.3 | syndecan |
| T5-Ca_V-UP and T5-Mi_V-UP | 1 | XM_019706142.3 | uncharacterized LOC109430111 |
| T5-Ca_C-UP and T5-Mi_V-UP | 2 | XM_029859167.2 | alpha-taxilin |
|  |  | XM_062856480.1 | zinc finger protein 260 |

**Down-regulation of immune families upon viral challenge**

Fig C displays mirrored bar graphs representing the percentage of contigs within each immune group that were at least twice as abundant in control samples compared to infected samples, based on FPKM counts.


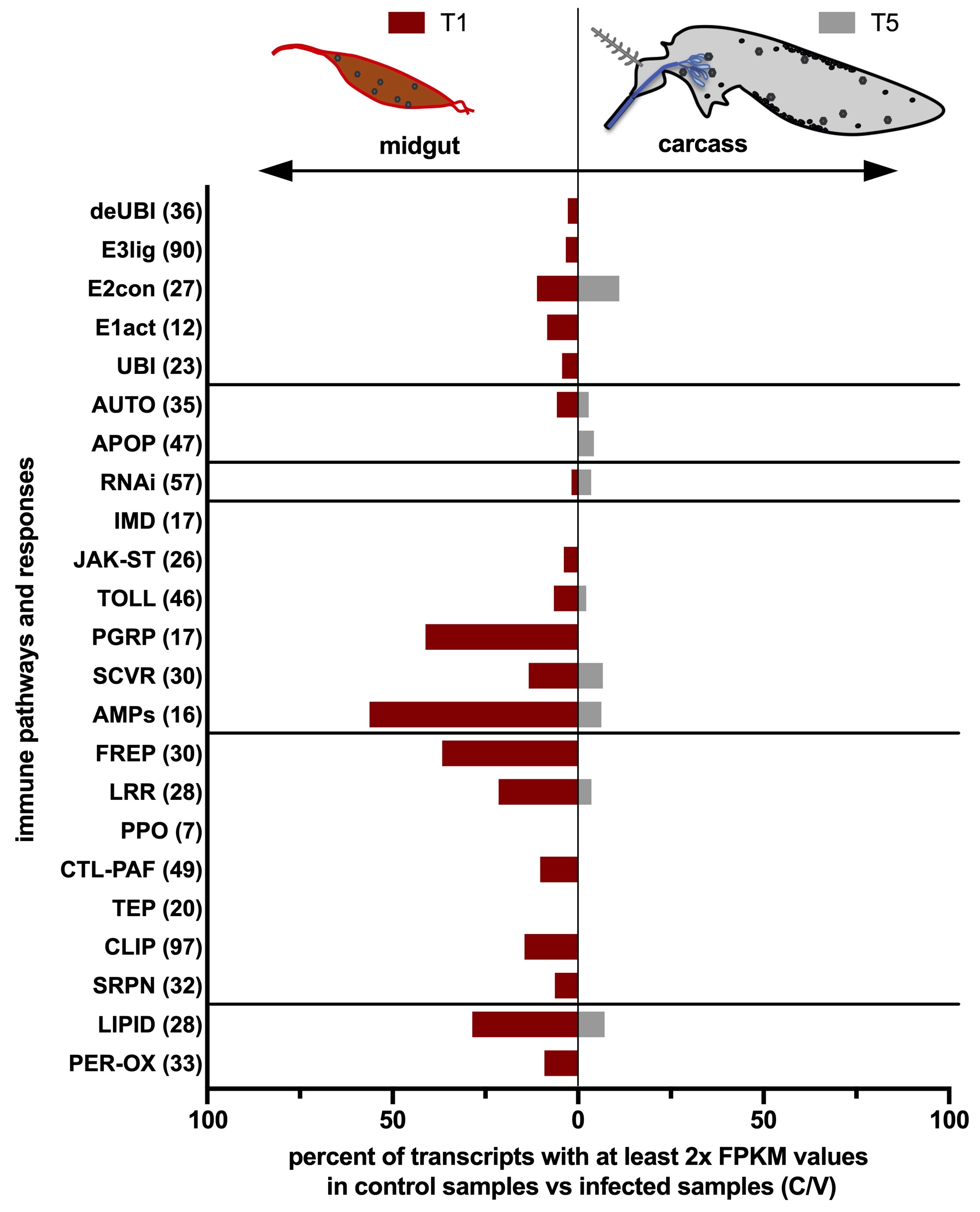


**Fig C. Reduced transcriptional expression of immune gene family members following viral challenge.** A mirrored bar chart illustrates the percentage of transcripts from each immune group (listed on the y-axis, with the number of gene family members in brackets) that were at least twice as abundant in control samples compared to infected samples, based on FPKM values. The x-axis shows these percentages: red bars on the left represent data from midguts at 1 dpi, while grey bars on the right correspond to carcasses at 5 dpi.

**Primers’ sequences**

**Table D. List of primers.**

| **Primer** | **Sequence** | **Primer** | **Sequence** |
| --- | --- | --- | --- |
| SP_QF | ATGCTTCCGAGTTCCAGGTG | SP_QR | TACGACGGATGAGGCCAAAC |
| HOLO_QF | GCACGGAGGAAAACAAGGTT | HOLO_QR | CCGTGCTTCTTGTATCCGTG |
| LRR_QF | AAACATTTGCTGCACTCCGG | LRR_QR | GCGTCTGAAAATCCGACTGC |
| PAF2_QF | TGCTCTCAGAACTACCAGGC | PAF2_QR | AAGTGTCGATGCCTTCCTCA |
| 013336_S5_QF | GTGCCACCGTGCTATAAAGTCCGT | 013336_S5_QR | ACGCCTTGACCACATGCTGAACGT |
| CHIKV for | TGATCCCGACTCAACCATCCT | CHIKV rev | GGCAAACGCAGTGGTACTTCCT |
| ChikProbe | FAM-TCCGACATCATCCTCCTTGCTGGC-Black Hole Quencher 1 | | |

**Comparison with previous transcriptomic datasets**

In the study published by Modahl and colleagues, authors applied transcriptomic analysis to profile genome-wide responses of *A. albopictus* to CHIKV and of *A. malayensis* to CHIKV and DENV at 1 and 4 days post-infection (dpi) in midgut tissues. In *A. albopictus*, CHIKV infection significantly altered the expression of 1,793 genes at 1 dpi and 339 genes at 4 dpi. When we compared the 1,793 DEGs from the Modahl database (1,010 upregulated, 783 downregulated) with the 145 DEGs from our database (73 upregulated, 72 downregulated), we found that 17.2% of the DEGs in our dataset were also present in the Modahl database (Supplementary S7 Data File). Gene descriptions and Pfam IDs were retrieved from the Foshan FPA database available in VectorBase (Supplementary S7 Data File). Several key contigs and Pfam domains identified in our transcriptome analysis were common to both datasets. Among the genes differentially upregulated by CHIKV in the midgut at 1 dpi, we found E3-ubiquitin protein ligase, DE-cadherin, protein bark beetle, and peroxisomal oxidase, along with domains such as scavenger receptor, EGF-like, and alpha-2-macroglobulin. Conversely, among the genes downregulated at 1 dpi, common hits included lipase, carboxypeptidase, trypsin, and long-chain-fatty-acid–CoA ligase. These overlapping results strongly support the importance of certain pathways in early midgut–virus interactions. Although the percentage of shared DEGs between the two databases was relatively low (17.2%), their expression profiles showed a strong degree of coherence. To further assess this, we used DEG of the Modahl dataset (1,793 DEGs) as query and extracted the corresponding expression values (FPKM) and fold change values (log2FC, infected vs. non-infected midguts at 1 dpi) from our transcriptome, harmonizing the datasets for comparison. The initial search yielded 2,370 contigs, which we filtered by retaining only those with at least one sample (infected or non-infected midguts) showing FPKM > 1 (1,360 contigs). After removing redundant contigs (isoforms, spliced or partial forms arising from differences in assembly), we obtained 679 unique transcripts shared between the two datasets. Analysis of the log2FC values for these 679 genes revealed a highly significant correlation between the two databases (Fig D), demonstrating strong overall reproducibility across both studies.


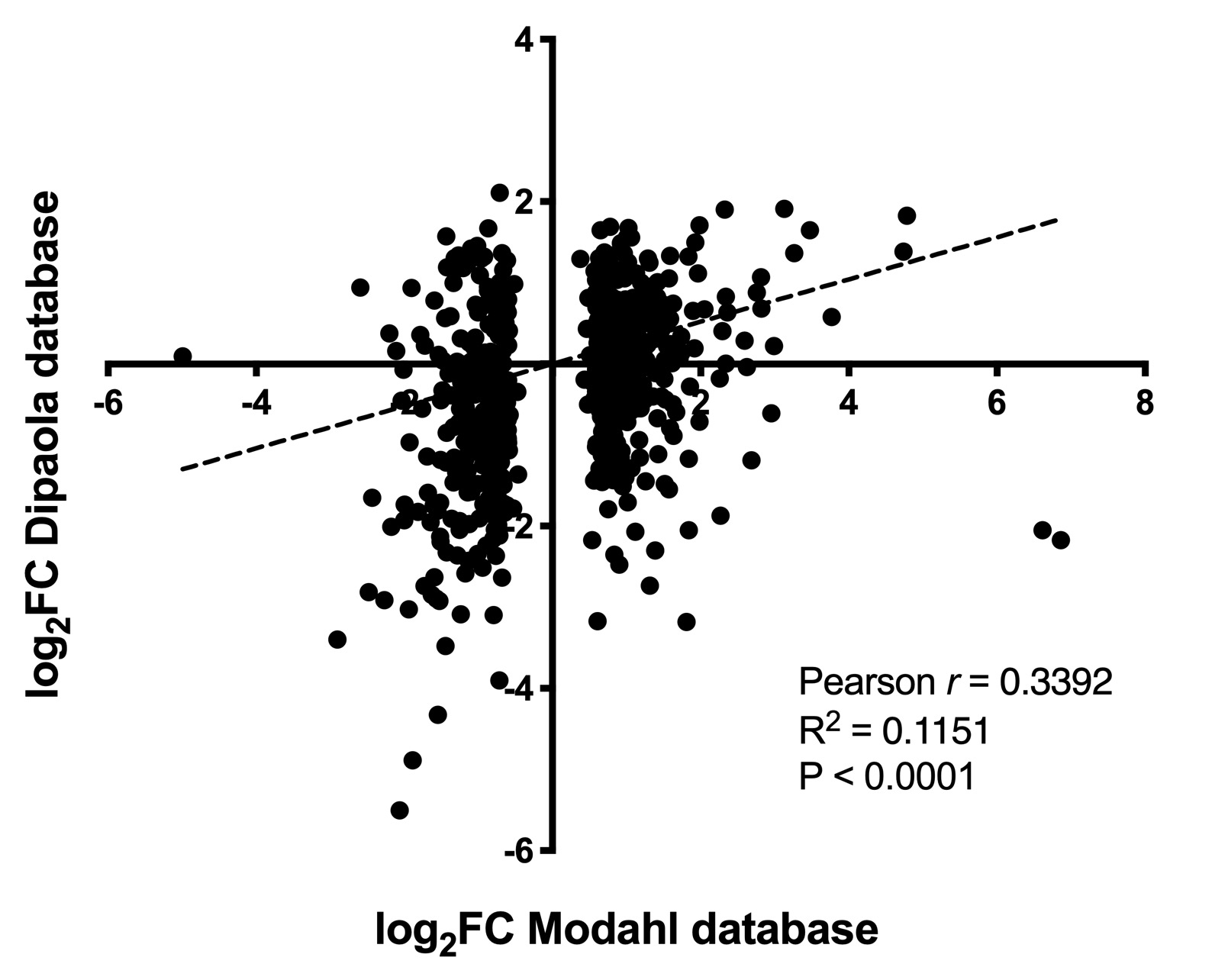


**Fig D. Correlation of differentially expressed genes (DEGs) between Modahl and Dipaola T1_Mi databases.**

Scatter plot comparing log₂ fold change (log₂FC, infected/not infected) values of 679 common contigs from the Modahl and Dipaola datasets. Each dot represents a contig; the dashed line indicates the linear regression fit. A significant positive correlation was observed (Pearson r = 0.3392, R² = 0.1151, P < 0.0001), indicating overall consistency in gene expression profiles between the two studies.

The second dataset from Modahl and colleagues examined transcriptional modulation in CHIKV-infected versus uninfected midguts at 4 dpi, differing from our dataset, which focused on midguts at 5 dpi. Nevertheless, we carried out a comparison between the two. Among the 339 DEGs identified in the Modahl T4_Mi dataset (262 upregulated, 77 downregulated) and the 152 DEGs in the Dipaola T5_Mi dataset (106 upregulated, 46 downregulated), 2.6% of the DEGs from the Dipaola dataset were also present in Modahl’s data (Supplementary S7 Data File). Gene descriptions and Pfam IDs were retrieved from the Foshan FPA database in VectorBase (Supplementary S7 Data File). Although the proportion of shared DEGs between the two datasets was very low (2.6%), their expression profiles still showed a strong degree of consistency. To further evaluate this, we used the 339 DEGs from the Modahl dataset as queries and extracted the corresponding expression values (FPKM) and fold-change values (log2FC, infected vs. non-infected midguts at 1 dpi) from our transcriptome (T5_Mi), thereby harmonizing the datasets for direct comparison. This search initially identified 545 contigs, which were filtered by retaining only those with at least one sample (infected or non-infected) showing FPKM > 1 (245 contigs). After removing redundant sequences (isoforms, spliced or partial forms due to differences in assembly), 132 unique contigs remained common to both datasets. Comparative analysis of log2FC values for these contigs revealed a highly significant correlation (Fig E), confirming the strong reproducibility of transcriptional profiles across the two studies.


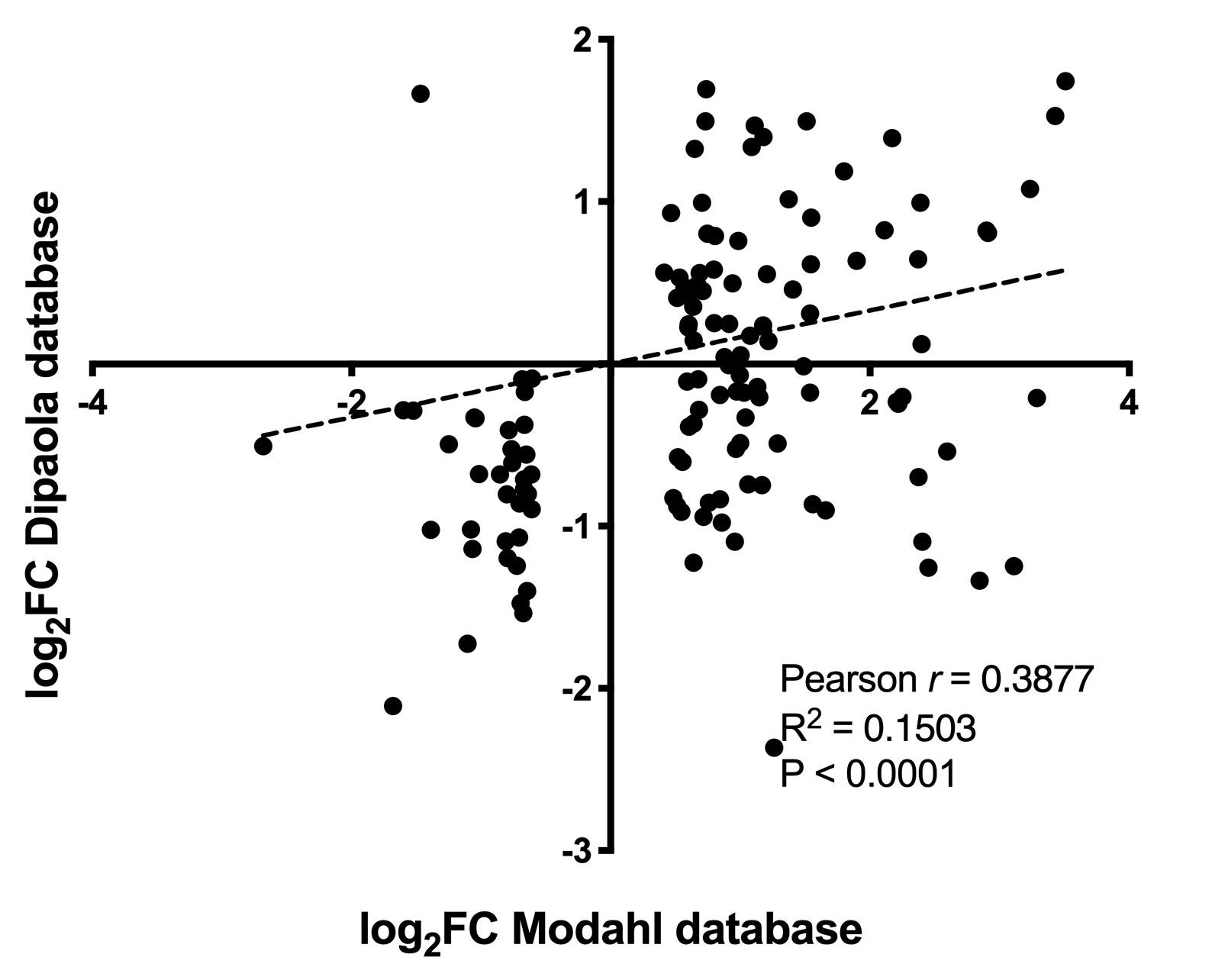


**Fig E. Correlation of differentially expressed genes (DEGs) between T4_Mi Modahl and T5_Mi Dipaola databases.** Scatter plot showing the correlation of log₂ fold change (log₂FC, infected/not infected) values for 132 differentially expressed genes (DEGs) common to both the Modahl (T4_Mi) and Dipaola (T5_Mi) transcriptomic datasets. Each dot represents an individual contig; the dashed line indicates the linear regression fit. A significant positive correlation was observed (Pearson r = 0.3877, R² = 0.1503, P < 0.0001), confirming a consistent expression trend across the two independent analyses.
